# Supplementary material for: A vibrating ingestible bioelectronic stimulator modulates gastric stretch receptors for illusory satiety
Source: Sci Adv. 2023 Dec 22;9(51):eadj3003. doi: 10.1126/sciadv.adj3003 (PMC10745699; doi:10.1126/sciadv.adj3003)
Supplement: 20231222-1 [file sciadv.adj3003.v1.pdf]

ENGINEERING

Copyright © 2023 The Authors, some rights reserved; exclusive licensee American Association for the Advancement of Science. No claim to original U.S. Government Works. Distributed under a Creative Commons Attribution License 4.0 (CC BY).

A vibrating ingestible bioelectronic stimulator modulates gastric stretch receptors for illusory satiety

Shriya S. Srinivasan[1,2,3,4]*, Amro Alshareef[1,2,3], Alexandria Hwang[1,2], Ceara Byrne[1,2], Johannes Kuosmanen[3], Keiko Ishida[2,3], Joshua Jenkins[1], Sabrina Liu[3], Wiam Abdalla Mohammed Madani[3], Alison M. Hayward[1,2,5], Niora Fabian[1,3,5], Giovanni Traverso[1,2,3]*

Effective therapies for obesity require invasive surgical and endoscopic interventions or high patient adherence, making it challenging for patients with obesity to effectively manage their disease. Gastric mechanoreceptors sense distension of the stomach and perform volume-dependent vagal signaling to initiate the gastric phase and influence satiety. In this study, we developed a new luminal stimulation modality to specifically activate these gastric stretch receptors to elicit a vagal afferent response commensurate with mechanical distension. We designed the Vibrating Ingestible BioElectronic Stimulator (VIBES) pill, an ingestible device that performs luminal vibratory stimulation to activate mechanoreceptors and stroke mucosal receptors, which induces serotonin release and yields a hormonal metabolic response commensurate with a fed state. We evaluated VIBES across 108 meals in swine which consistently led to diminished food intake (~40%, $P < 0.0001$) and minimized the weight gain rate ($P < 0.05$) as compared to untreated controls. Application of mechanoreceptor biology could transform our capacity to help patients suffering from nutritional disorders.

INTRODUCTION

The obesity epidemic, affecting nearly 42% of US adults (*1*, *2*), increasingly strains healthcare resources by increasing the incidence of comorbidities such as diabetes, hypertension, cancer, and heart disease (*3*, *4*). Given the difficulty of modifying behaviors and limitations of pharmacologic therapies, there remains a pressing need for alternative methods that efficaciously decrease weight gain. While bariatric surgeries have demonstrated efficacy and have evolved as minimally invasive laparoscopic procedures (Roux-en-Y and laparoscopic banding), they require extensive pre- and post-surgical lifestyle modifications and remain too costly ($7400 and $34,000) for the global populations requiring treatment (*5*–*7*).

Vagal nerve signaling plays a critical role in satiation through a negative feedback loop in which anorexigenic neurometabolic secretions are released in response to food intake (*8*). Distension of the stomach by food contents is transduced by intraganglionic laminar endings (IGLEs), the most prevalent type of vagal afferents innervating the gastric musculature, which sense contraction and distension (*9*, *10*). These stretch mechanoreceptors produce short-acting vagal afferent signals and increase neuronal activity in the nucleus of the solitary tract (NTS) (*11*) where vagal afferents terminate and interact with reward, energy homeostasis, hunger, and mood circuitry (*9*, *12*). In turn, the NTS triggers metabolic and neural anorexigenic signaling (*13*–*15*) to modulate feelings of hunger or fullness and alter food intake (*16*). Because this mechanism is primarily volume dependent (*17*), as opposed to composition dependent [carbohydrates, proteins, fats, or saline (*18*)], methods to manipulate gastric volume, including intragastric balloons (IGBs), were developed as an easy-to-deploy tool (*19*) to minimize weight gain.

IGBs are designed to induce stomach distension to induce early satiety. Although they enable short-term weight loss during the adaptation phase, IGBs fail to promote sustained changes in hunger or eating behavior after 10 to 12 weeks (*20*) and do not demonstrate superior outcomes compared to pharmacologic or surgical therapy (*19*). Neural adaptation to the chronic distension (as opposed to periodic distension that results from eating), as well as placement, removal, perforation, and obstruction complications pose challenges for the long-term efficacy and safety of IGBs (*21*, *22*). Following numerous deaths in patients with IGBs since 2016, the Food and Drug Administration (FDA) has issued warnings and some companies have recalled their IGB products (*23*).

Intervening more proximally, vagotomies and electrical vagus nerve stimulation (VNS), which are localized interventions, have been preclinically demonstrated to be associated with decreased weight gain, food intake, and sweet cravings and increased satiation and energy expenditure (*12*). When clinically implemented for depression and epilepsy, VNS has shown a decrease in weight gain, reduced sweet cravings, and increased energy expenditure. However, non-gastrointestinal (GI) side effects resulting from non-specific axonal targeting at the cervical level of the vagus and metabolic compensatory mechanisms have prevented widespread clinical implementation for obesity (*24*). Vagotomies have also demonstrated considerable benefit (*25*), although the mechanism and side effects are unclear and involves an invasive surgical procedure (*26*). Fundamentally, with our current technology and understanding of neural signaling, VNS systems cannot specifically target the relevant axons nor perform patterned stimulation to recapitulate the complex physiological signaling underlying satiety.

[1]Department of Mechanical Engineering, Massachusetts Institute of Technology, Cambridge, MA 02139, USA. [2]Division of Gastroenterology, Hepatology and Endoscopy, Brigham and Women's Hospital, Harvard Medical School, Boston, MA 02115, USA. [3]David H. Koch Institute for Integrative Cancer Research, Massachusetts Institute of Technology, Cambridge, MA 02139, USA. [4]Society of Fellows, Harvard University, Cambridge, MA 02138, USA. [5]Division of Comparative Medicine, Massachusetts Institute of Technology, Cambridge, MA 02139, USA.
*Corresponding author. Email: cgt20@mit.edu, ctraverso@bwh.harvard.edu (G.T.); shriya_srinivasan@fas.harvard.edu (S.S.S.)

Considering the central role of gastric mechanotransducers in vagal neurometabolic satiety signaling (*27*), a mechanism and/or devices capable of selective mechanoreceptor activation would pose great clinical value. Seminal experiments in stretch-sensitive spindle fibers in skeletal muscle have shown that vibration elicits illusory distension (*28*–*30*). Following a parallel mechanism, we devised a vibratory stimulation modality to selectively activate gastric stretch receptors and characterize their response in this proof-of-concept study. We hypothesized that optimized intraluminal vibration of the gastric smooth muscle would induce illusory distension of the stomach, generating vagal afferent signals and a metabolic response commensurate to those elicited from mechanical distension in a fed state (Fig. 1A). We then developed the Vibrating Ingestible BioElectronic Stimulator (VIBES) pill, a safe, easy-to-use, ingestible device that can perform temporary, targeted, intraluminal mechanical stimulation before meals to achieve early satiety. In awake and freely moving swine, we hypothesized that the VIBES would reduce food intake and minimize the rate of weight gain as compared to untreated controls.

RESULTS

Design and characterization of the VIBES pill

The VIBES was designed to be orally ingested, to sustain contact with the gastric lining, to activate upon submersion in gastric fluid, to vibrate with amplitudes sufficient to stimulate gastric IGLEs for a set time period, and to pass safely through the GI tract. Its triple-zero capsule houses a gelatinous membrane, which dissolves in 4.3 ± 1.2 min following immersion in gastric fluid, releasing a spring-loaded pogo pin that completes the circuit to activate the vibrating motor. Pill ingestion and travel through the esophagus is known to take generally a maximum of 60 s, ensuring that the pill will reach the stomach before activation (*31*). A motor with an offset shaft is positioned within a custom housing enabling displacement amplitudes of 2 to 4 mm when powered with a 1.55-V, 80-mA·hour silver oxide battery.

Duration testing was performed by immersing the VIBES pill in gastric fluid on a soft substrate. The pill actively vibrated for an average of 38.3 ± 1.83 min for the VIBES pill (n = 5). Because meals are generally consumed in a 20- to 30-min window and gastric contents undergo primary mixing in approximately an hour, this time range was determined to be acceptable. To ensure that the system did not degrade or have material vulnerabilities, a chemical resistance test (described in Methods) was performed by immersing the VIBES pill in simulated gastric fluid (pH 1.2) for 24 hours and simulated intestinal fluid for 10 days, both at 37°C. No macro- or microscale changes were observed following immersion, and pills were able to be successfully activated after the incubation period (fig. S1). Thus, the VIBES pill would not damage the GI tract even if it were to reside in the stomach for a full day or in the intestines for over a week, much longer than the expected or measured residence times with normal motility. Thermal testing was performed to assess any potential heating risks on the surrounding tissue environment. Thirty minutes of operation at various motor frequencies yielded less than a 0.5°C change in the surrounding fluid, ensuring no thermal risks for the mucosal layer during operation (fig. S2).

A swine model (50- to 80-kg Yorkshire pigs ranging between 4 and 6 months of age) was used to study the VIBES performance, as its gastric anatomy is similar to that of humans. Furthermore, it has been widely used in the evaluation of biomedical GI devices (*32*). Localization of the VIBES pill was characterized through endoscopic observation in n = 10 swine. VIBES was endoscopically deployed into the gastric cavity, and the final positioning was observed through the video channel over the course of 30 min. The VIBES pill exerts 0.04214 N of downward force and has a density of 2.019 g/cm^3. In all 10 trials, the pill sank through gastric contents (densities, 0.011 to 1.158 g/cm^3) and achieved stable contact with the lining (note S1). Localization in the gastric antrum and cardia were common and dependent on the positioning of the animal. In several cases, over the course of 30 min of stimulation, the pill was seen to migrate along the lining. During the 30-min period, trained endoscopists monitored the density of the rugae in the empty stomach to estimate changes to the gastric cavity volume. In all trials, no differences in the stomach volume were visualized.

In all cases, the VIBES was not emptied from the stomach by phasic interdigestive migrating motor complex for at least 30 min following administration supporting the potential for prolonged gastric residence for pre-prandial dosing for the distension repsonse.

Stretch-receptor activation-to-signal gastric distention

To characterize the neural signaling patterns of the stomach in response to mechanical distension and vibrational stimulation (n = 4 animals), fine-wire electrophysiological recordings of up to 24 branches of the celiac vagus nerve, which innervate the gastric cardia, were performed following laparotomy. The celiac vagus was selected to isolate signals arising from the stomach and mitigate off target signals from other organs. Baseline recordings demonstrated small-amplitude, spontaneous spikes along with slow wave–related spiking. To mimic the distension created by the intake of food, the stomach was inflated using the endoscope to 30, 60, and 90% of its maximal volume and held for 180 s (fig. S3). Five minutes of rest were provided between each insufflation state to allow neural activity to return to baseline. Spiking was observed in a subset of 6 to 10 channels with onset occurring 10 to 12 s following the beginning of inflation (Fig. 2, A and B); these channels were designated as channels corresponding to axons innervating low-threshold stretch-sensitive IGLEs, consistent with prior studies (*10*). Spiking amplitude was higher and more densely concentrated at the onset of stretch followed by a slow adaptation rate (leveling off of distention-based afferents as the stomach adapts), commensurate with prior reports of these afferents (Fig. 2B) (*10*). Upon desufflation, spiking acutely decreased and terminated within 2 to 18 s. The rate of firing graded monotonically with distension, which is commensurate with the known spiking patterns of IGLEs (Fig. 2A) (*10*). We modulated the distension by varying the volume of insufflation in the stomach.

To determine whether vibratory stimulation could activate these IGLEs to produce a similar response, neural activity on the channels that were active for stretch-sensitive IGLEs were monitored as vibration with frequencies between 24 and 500 Hz was applied using the VIBES to the gastric lumen (fig. S4). Although the pill provides a contact surface area of approximately 298 mm^2, the vibratory stimulation propagates through tissue, stimulating the gastric muscularis up to 30 cm away.

In all trials, spiking was observed at frequencies between 64 and 100 Hz in a similar pattern to those observed during mechanical

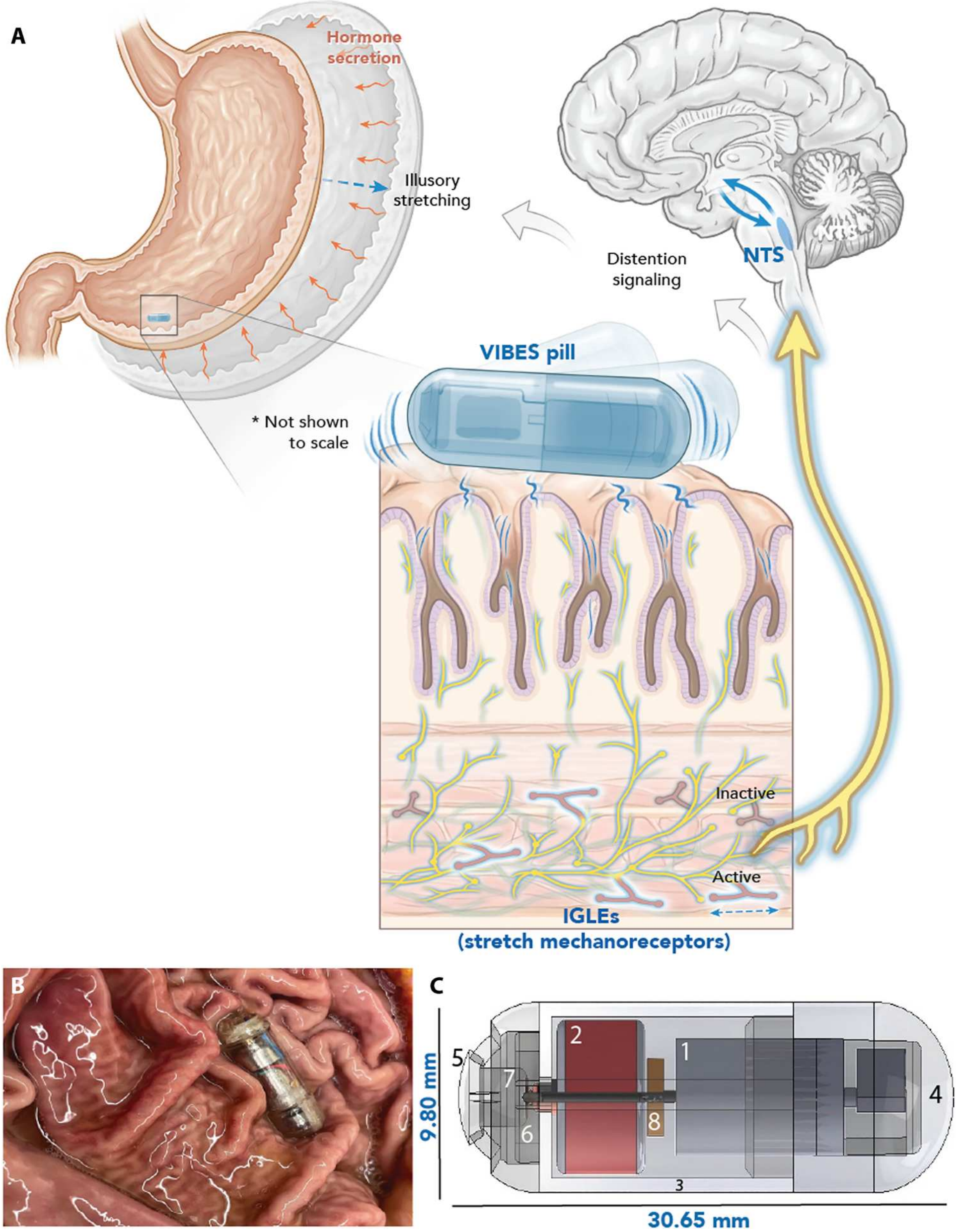

Fig. 1. VIBES concept and mechanism. (**A**) The Vibrating Ingestible BioElectronic Stimulator (VIBES) makes contact with the gastric lining and activates following contact with gastric fluid. Vibrations activate intraganglionic laminar endings (IGLEs) in the celiac plexus, signaling distension to the NTS, which interacts with hunger circuitry to signal illusory distension. (**B**) VIBES sits among gastric rugae in a swine stomach and strokes the mucosa as it performs stimulation. (**C**) The VIBES pill consists of an (1) offset motor, (2) silver oxide battery, (3) central body, (4) motor cap, (5) pill cap, (6) pogo pin, (7) gelatinous membrane, and (8) resistor. Original illustration by V. Fulford.

distention (Fig. 2C). At these frequencies, the amplitude of displacement of the pill was greater than 1 mm. No activation was observed for displacements under 1 mm or for frequencies below 35 Hz or above 150 Hz. Onset of activation occurred within 2.5 to 3.2 s. The change in signal amplitude demonstrated slow adaptation, similar to mechanically activated afferents (fig. S5). Bilateral vagotomy was then performed, and the above results were replicated to eliminate potential confounding between efferent signals and feedback loops. Raw spiking patterns from (Fig. 2B) mechanical insufflation (Fig. 2B), VIBES (Fig. 2C), and VIBES after bilateral vagotomy (Fig. 2D) demonstrate a high degree of similarity in terms of frequency spectra and coherence. In a comparison of frequency spectra between the signals generated in the control and VIBES groups (fig. S4), the dominant peak was identical in both groups at 2904 Hz with similar amplitudes. Furthermore, the five greatest peaks occurred at the same frequencies in both groups. Moreover, cross-spectral coherence between the VIBES and control afferents was 0.0224, which is not statistically different at a P value of 0.4426. A two-tailed t test of the average signals was also insignificant at an alpha of 0.1. Furthermore, spiking was able to be elicited repeatedly (Fig. 2E), without significant reduction in the onset or plateau amplitude ($P < 0.05$, $n = 10$). These results

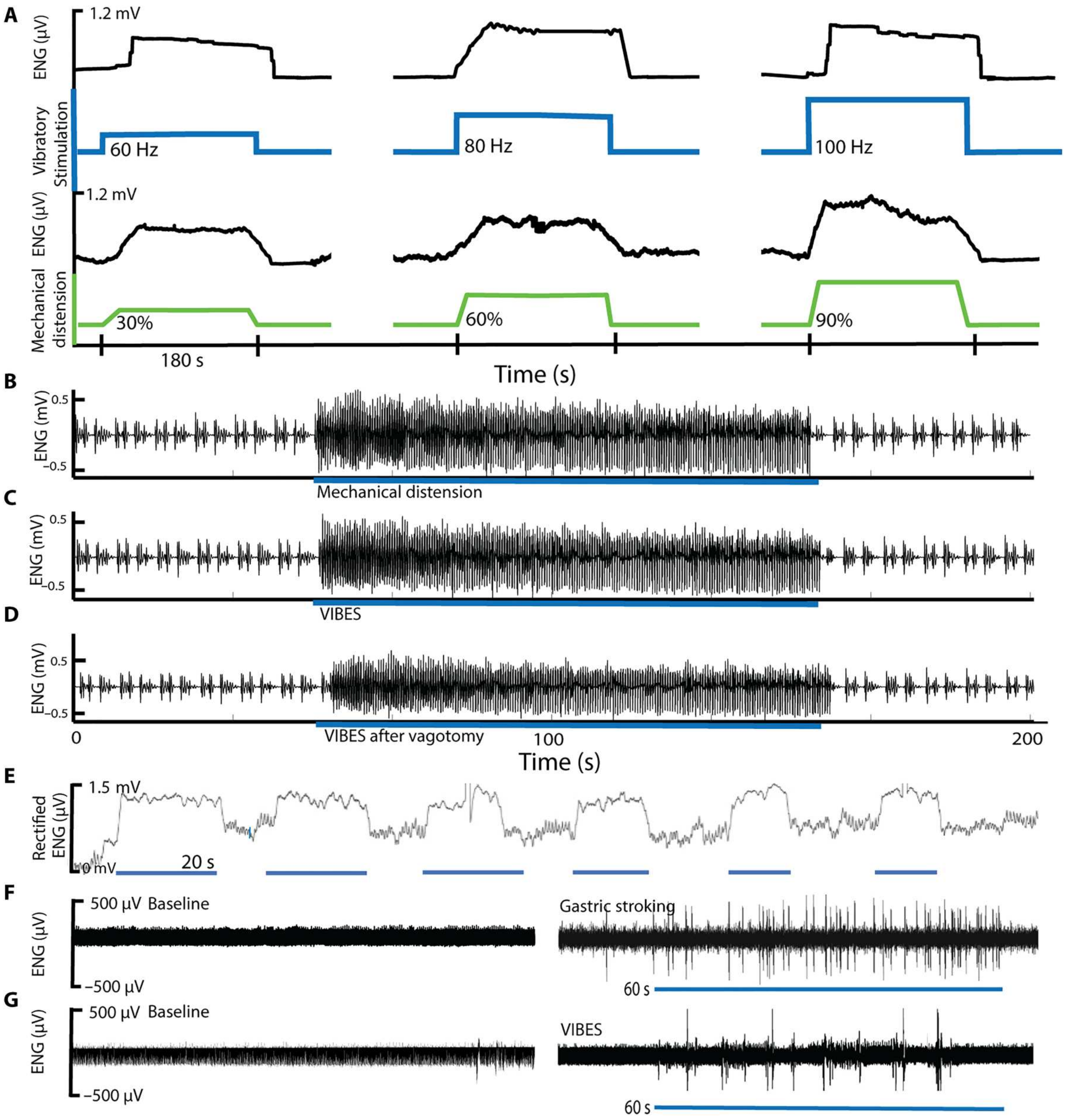

Fig. 2. Gastric afferent electrophysiology of stretch-sensitive mechanoreceptors. (**A**) The gastric cavity (green) was distended to 30, 60, and 90% of the gastric volume using insufflation. The corresponding electroneurographic (ENG) vagal response was recorded from celiac vagal branches demonstrating spiking in response to distension. Vibratory stimulation at 60, 80, and 100 Hz (blue) resulted in similar, monotonically graded ENG responses (black). ENG signals have been rectified for visualization. (**B** and **C**) ENG signal from a stretch sensitive afferent fiber responding to (B) mechanical inflation and (C) a Vibrating Ingestible BioElectronic Stimulator (VIBES) pill within the stomach and (**D**) a VIBES pill after bilateral vagotomy. Heart rate and respiratory artifact are present at baseline. (**E**) Periodic VIBES-mediated vibration resulted in repeatable induction of the stretch response. Afferent response to mucosal stroking of the gastric lumen by an (**F**) endoscopic fiber and (**G**) VIBES pill rotation against the mucosal surface. Blue bars underlying electrophysiological traces indicate when stimulation occurred.

suggest that gastric luminal vibration induces afferent neural activation of gastric mechanoreceptors sensitive to distention.

Gastric mucosal stroking

In addition to distension, mucosal stroking triggers gastric mechanoreceptors to stimulate gastric secretory activity. Gastric mucosal stroking was conducted via an endoscope using a thin filament that is known to elicit spiking for such receptors (Fig. 2F). During VIBES treatment, in axons not activated by stretch, we observed such periodic bursting (Fig. 2G). The surface geometry of the VIBES pill may have stroked the gastric mucosa as it rotated, resulting in short, periodic bursts likely from quickly adapting mucosal receptors (*11*, *33*). Mucosal stroking is known to release 5-HT or serotonin (*34*–*36*), which acts on vagal 5-HT_3 receptors that perform satiation signaling and enteric 5-HT_4 receptors, which regulate peristalsis, secretion, vasodilation, and digestion through intrinsic central and peripheral reflexes. Given the electrophysiological results, we assayed the luminal secretion of serotonin in response

to VIBES using ex vivo tissue on a Franz cell apparatus (*37*). Eighty hertz resulted in the greatest increase in 5-HT secretion levels as compared to the control condition of 0 Hz (fig. S6). On the basis of this data and the inconvenient human audibility of the VIBES pill above 100 Hz, 80 Hz was selected as the optimal operating frequency. In addition, surface geometries were altered to enable greater stroking of the mucosa (fig. S7), including studs and ridges to enhance surface contact. A spiraling design induced a significant increase in luminal serotonin release as compared to the straight surface and control conditions ($P < 0.05$, Student's two tailed t test).

Metabolic effect of VIBES

To characterize the downstream effects of VIBES through vagal afferent signaling, we profiled the hormonal secretions relevant to feeding behavior and satiety. Blood was sampled at 0, 15, 30, 45, 60, 90, and 150 min for animals receiving a VIBES pill ($n = 6$, between the 30th to 60th minute marks) and a sham pill ($n = 6$). The effect was ascertained using a two-tailed heteroscedastic t test of the average value of the hormone after stimulation between the control and VIBES groups at an alpha of 0.05 (Fig. 3). After stimulation, the pill remained in the tract and was allowed to pass naturally. While the control animals exhibited the expected levels of these hormones in a fasted state, treatment with VIBES resulted in a significant reduction of ghrelin, the "hunger hormone" ($P < 0.01$). Ghrelin decrease is normally a postprandial response but occurred here in fasted animals treated with VIBES (Fig. 3A). Furthermore, insulin levels increased significantly ($P < 0.01$) with an amplitude and rate commensurate with normal meal ingestion (Fig. 3B). Glucagon, responsible for maintaining blood glucose levels, increased in control animals, while it remained at baseline in the VIBES group (Fig. 3C). Levels of C-peptide, associated with the biosynthesis of insulin, GLP-1, which enhances insulin secretion, and Pyy, an appetite suppressant, increased significantly with stimulation ($P < 0.01$; Fig. 3, D to F). Together, these trends suggest that by signaling distention artificially, VIBES can induce the gastric phase metabolic response. Glucose from a venous catheter was measured at 30-min intervals before and after administration of VIBES. No significant change was present between groups ($P < 0.01$; Fig. 3G). No animals demonstrated hypoglycemia during the course of the study.

VIBES reduces food intake

To investigate the effect of VIBES on hunger and feeding behavior, the food intake of four swine was monitored for at least 24 meals with no treatment (control group) and when treated with VIBES tethered through a percutaneous endoscopic gastrostomy (PEG) tube (VIBES group). Four animals matched in size and age with a PEG tube were also monitored (PEG-control group), to account for potential effects of the PEG tube. Tethering was performed with a very flexible tether of 10 to 15 cm, enabling the VIBES to excurse around the stomach freely. During endoscopic observation, the

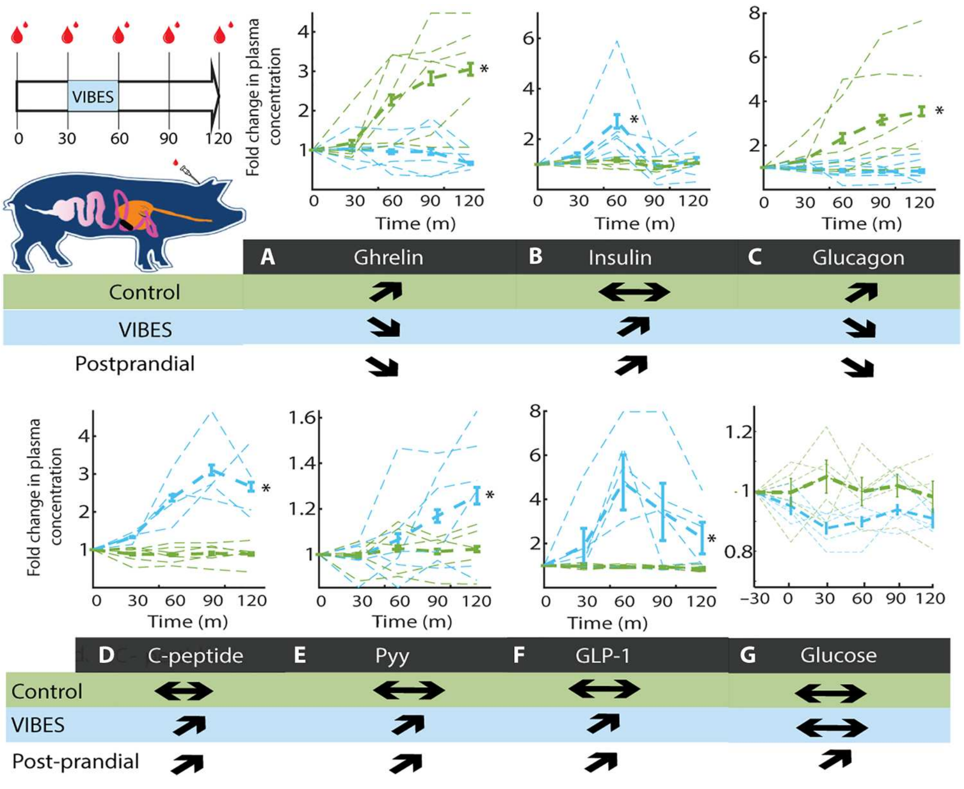

Fig. 3. VIBES neuromodulation of gastric vagal afferents yields illusory metabolic satiety. (**A**) Experimental schematic for blood sampling during Vibrating Ingestible BioElectronic Stimulator (VIBES) stimulation between 30 and 60 min. (**B** to **G**) Hormonal response normalized to their baseline levels in animals with no stimulation (green) and VIBES (blue). Individual subjects' traces are shown with the group average in bold. Arrows indicate the observed trends for the control and VIBES groups and represent the known trend in animals and humans postprandially. Stars indicate significance in difference of the area under the curve at $P < 0.05$ (Student's two-tailed t test).

tethered VIBES demonstrated no difference in contact, vibration, mechanical force transmission, and/or stroking of the gastric lining as compared to a free-floating capsule. Stimulation was performed for 30 min before mealtimes at 7:30 a.m. and 3:30 p.m. when the food hopper would be filled with pellets. In addition, a snack of five apples was provided between 11 a.m. and 12 p.m. While animals were fed ad libitum, the proportion of food consumed in the first 30 min after provision was measured to be the intake for a given meal.

Prior studies have demonstrated that satiety is well marked by the amount of food consumed in animals (*38*).The average percentage and SD of the meal consumed for the VIBES, PEG-control, and control group were 58.1 ± 10.7 (n = 108 meals), 84.1 ± 4.5 (n = 100 meals), and 78.4 ± 4.5 (n = 96 meals), respectively. Intake in the VIBES group was significantly lower than the PEG-control and control groups ($P < 10^{-20}$, Student's two-tailed homoscedastic t test; Fig. 4A). There was no significant difference between the PEG-control and control groups ($P > 0.05$), indicating that the presence of a PEG tube itself did not confound the significant difference seen during VIBES treatment. The distributions in the intake of each group were compared using the Kolmogorov-Smirnov test to further characterize the effect of the VIBES intervention. Between the VIBES and control groups, the P value of 6.0103×10^{-7} indicates that the distributions are significantly different with a maximal vertical distance between the cumulative distribution functions (CDFs) of 0.4232. However, between the PEG-control and control groups, a P value of 0.055 and maximal distances between CDFs of 0.2119 indicate that there is no significant difference between these distributions (fig. S16). On a per-animal basis, VIBES treatment resulted in significant reductions in intake ($P < 0.001$ in all cases, Student's two-tailed homoscedastic t test), averaging 31% of the usual intake. Energy consumed per meal by each animal treated with the VIBES was also significantly lower than those of the control group ($P < 0.001$ in all cases, Student's two-tailed homoscedastic t test; Fig. 4C). However, no significant difference in energy consumed was present between the PEG-control and control groups ($P < 0.1$, Student's t test). No adaptations or trends in intake were observed over the 24-meal period. To assess latencies and potential long-lasting effects of the treatment, a cross-over study design was used in which swine were treated for three meals, untreated for the subsequent three, and treated for the final three (Fig. 4D). Intake sharply increased 38% during the untreated window (average percentage intake, treated = 51% and untreated = 89%), suggesting that VIBES functions through temporal vagal activation, with little neural adaptation or long-term effect. We also analyzed the trend of intake over the course of the study for each subject using Pearson's correlation coefficients. No animals in any group demonstrated a significant trend in intake pattern (alpha = 0.1). This further indicates that there is no habituation or adaptation to the VIBES treatment. The weight gain rate during the VIBES treatment period was significantly lower than the control period (VIBES versus PEG-control: $P < 0.03$, Student's two-tailed paired t test; Fig. 4E) and significantly lower than the control group (VIBES versus control: $P < 0.01$, Student's two-tailed heteroscedastic t test). Together, these data suggest that the VIBES pill significantly decreases food intake and slows the rate of weight gain in a large animal model.

A preliminary behavioral study was conducted using an image-based deep learning model (accuracy of 94%) trained on 12 continuous hours of labeled daytime data (7 a.m. to 8 p.m.). This was used to analyze 96 hours of unlabeled daytime video data from four pig subjects in either the VIBES treatment or PEG-control conditions. We analyzed the time spent within each of the four behaviors, detailed in Table 1 in terms of occurrences, durations, and probabilities within the control and treated conditions. For all four behaviors (active, feeding, drinking, and inactive), the VIBES and PEG-control conditions were significantly different [F, $P < 0.001$, two-way analysis of variance (ANOVA); Fig. 4F]. Interaction effects were insignificant ($P < 0.01$), indicating that the response to each behavior was independently significant. Notably, the VIBES group spent significantly less time engaged in active behavior. Such reductions in physical activity and foraging have been reported to reflect postprandial satiety in laboratory pigs (*39*). As animals treated with VIBES demonstrated a postprandial metabolic state and consumed less food on average as compared to PEG-controls, we hypothesized that they may demonstrate more inactive behaviors after mealtimes, corresponding with a higher drop in blood glucose levels (*39*, *40*). We thus categorized behavior on an hourly basis (Fig. 4, G and H). On an hourly basis, the VIBES and PEG-control conditions were significantly different (F, $P < 0.001$, two-way ANOVA). Furthermore, probability of inactive behavior was significantly higher in the VIBES group, especially after meals ($P < 0.001$ at 2 p.m., 5 p.m. and $P < 0.01$ at 7 p.m.).

Safety and biocompatibility

A blinded endoscopist assessed the lining of the stomach and surveyed for inflammation, degeneration, morphological changes, and other potential adverse effects from the pill in control (n = 6) and VIBES animals (one administration of 30-min stimulation) (n = 6)

Table 1. Behavior classification.

General category	Behavior type	Behavior description
Inactive	Sleeping	No movement, not standing, eyes open
	Resting	No movement, not standing, eyes closed
Feeding	Free feeding	Head is in food bin, chewing
	Pedialyte	Pedialyte through cage doors while in vivo team exchanges batteries/fixes bandages: head touching bottle
Drinking	Water spigot	Head to water spigot
Active	Hanging toy ball	Head or body touching the hanging ball
	Hanging gear	Head or body touching the gears
	Free-moving toy	Interaction with head/ body; moving
	Scratching/rooting woodchips	Pawing at or using their nose to move woodchips
	Scratching or climbing on walls of cage	Body up and touching the walls of the cage
	Interaction with researcher	Touching, nosing, being fed from researcher

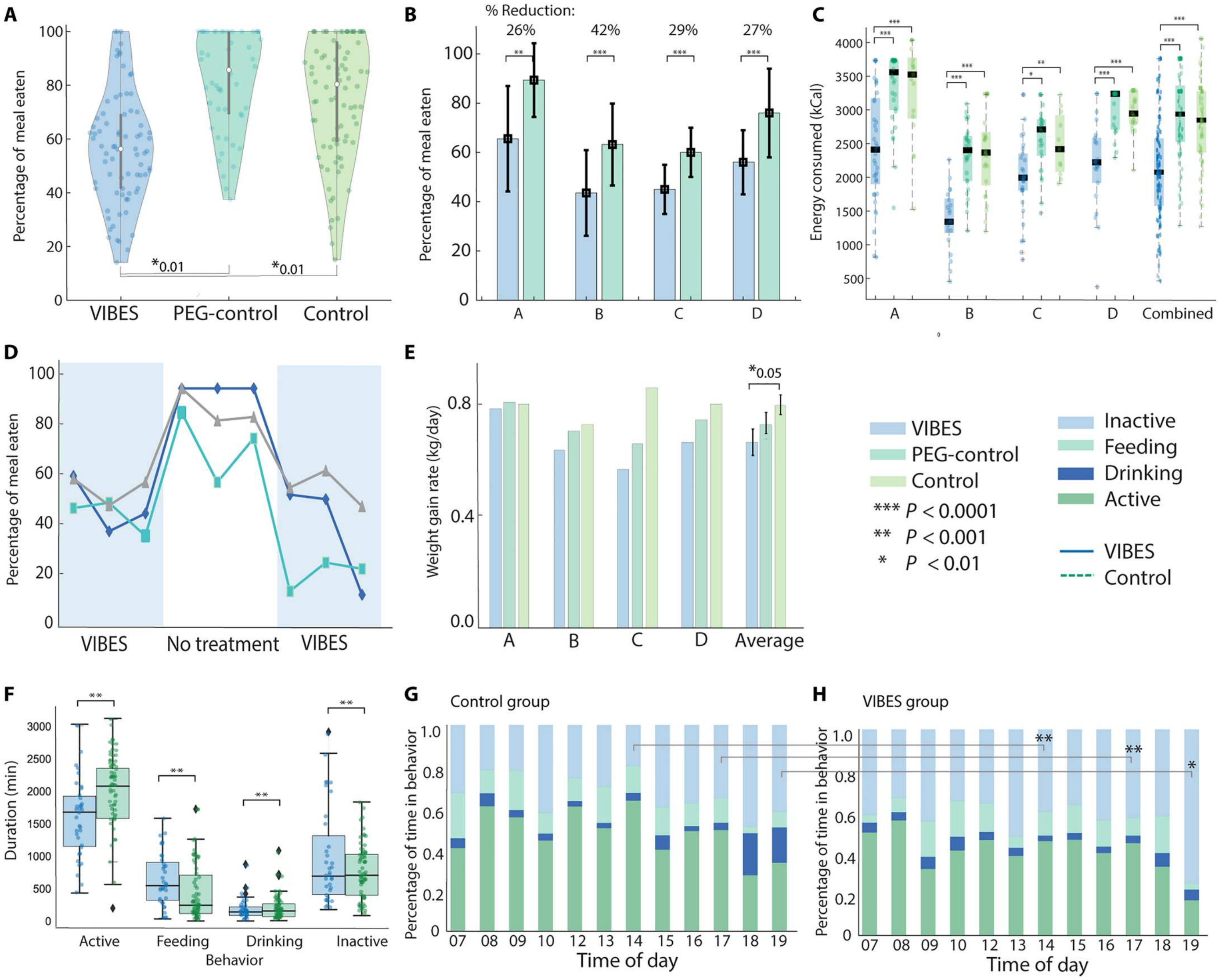

Fig. 4. VIBES effect on feeding and weight gain. (**A**) Percentage of meal consumed by swine in the Vibrating Ingestible BioElectronic Stimulator (VIBES), percutaneous endoscopic gastrostomy (PEG)–control, and Control groups. (**B**) Percentage of the meal consumed by animals A, B, C, and D over 2 weeks treated on VIBES and 2 weeks with no treatment (control). (**C**) Energy consumed at each meal (dots) by animals A, B, C, and D over 2 weeks treated with VIBES, PEG-control, or no treatment (control). Bars represent median and quartiles. (**D**) Consumption of animals A (green), B (gray), and C (blue) in a cross-over study design comprising three meals with VIBES treatment, no treatment, and VIBES, consecutively. (**E**) Weight gain rate for each animal when treated with VIBES was significantly lower than with no treatment in PEG-control and control groups ($P < 0.05$). (**F**) Duration of time that animals spent in each category of behavior. Diamonds indicate outliers. Percentage of time spent in each behavior for the (**G**) control group and (**H**) VIBES group.

during endoscopy. No abnormalities were noted in either group. Even after 2 weeks of daily VIBES usage (twice a day), no abrasion, irritation, or inflammation was observed on endoscopic examination of the gastric cavity (movies S1 and S2). Furthermore, hematoxylin and eosin staining on fixed sections of tissue following explant revealed no aberrant morphology, irritation, or inflammation (fig. S8).

To assess potential effects on motility and passage safety, animals were either fed a sham pill (n = 3 swine) or the VIBES pill (n = 3 swine) along with a set of small radio-opaque barium pellets. Radiography was performed every 2 days to determine the period required for clearance of all barium pellets (fig. S9). In animals with VIBES, swine passed all the pills in 4.3 days on average (range, 4 to 5 days), while in control animals, passage of a sham pill required 8.3 days (range, 7 to 9 days) (table S1). Furthermore, throughout the course of all trials in this study, veterinary staff monitored for changes in intake, health status, fecal output, lethargy, bloating, and other behavioral signs of discomfort or disease daily. No adverse effects or discomfort was observed in the treated animals. No changes in stool quality or diarrhea were observed in any animals in the experimental or control groups. These data suggest that VIBES does not have any negative impact on motility. In all trials, animals were able to pass the pill without obstruction, perforation, or any signs of distress.

The pH of gastric fluid was monitored using an ex vivo Franz cell apparatus to assess whether the VIBES created any significant shifts in the gastric fluid. Following 20 min of stimulation in the experimental group, the pH of gastric fluid was not significantly different from that of the untreated control group using a Student's two-tailed heteroscedastic *t* test ($n = 6$ samples per group). The mean and SD of the pH in the control and experimental groups was 1.265 ± 0.03 and 1.260 ± 0.029, respectively. As demonstrated by electrophysiology and the 5-HT response, the VIBES likely activates 5-HT_3 receptors, which trigger critical GI functions such as pancreatic secretion, meal termination, early satiety, and appetite regulation.

However, it is known that excessive activation of 5-HT_3 results in nausea and vomiting (*41*). Thus, all animals were monitored during treatment by four to six staff periodically during the day and through continuous daytime and nighttime video recordings 24 hours of the day. No signs of distress or emesis or diarrhea were observed in any animal.

DISCUSSION

In this study, we established a modality of luminal vibratory stimulation that activates gastric stretch receptors to signal distension and initiate the gastric phase. By optimizing the range of vibrational frequencies and including features in the VIBES pill that increased mucosal interactions, we not only generated vagal afferents signals relevant for indicating distention but also induced a significant and consistent decrease in food intake in swine. Restriction of caloric intake during meals is a well-documented and sustainable mechanism to limit weight gain. We envision the VIBES pill being ingested on a relatively empty stomach 20 to 30 min before anticipated meals to trigger the desired sensation of satiety early in the meal. Shaping this luminal stimulation modality into a pill format presents several valuable advantages over its alternatives. As an ingestible device, no invasive implantation or surgery is required. Stimulation can be performed directly in the gastric cavity with a triggered activation, making the stimulation specific to the tissue of interest.

At scale, with injection molding techniques and mass manufacturing of electronics, the cost is expected to be in the cents to dollar range. Coupled with natural passage, this makes the VIBES a consumable device, requiring no reacquisition or recharging of the device. For certain patient populations, this enables temporary therapy, without the need for surgery. However, with improved power transfer and charging technologies, implantable or gastric-resident actuators could be developed to relegate the need for repeated oral administration for patients requiring chronic therapy.

The robust design of the VIBES overcomes practical limitations common to human oral consumption/administration. The 30-Hz range of frequencies within which the stretch response is evoked is critical in enabling the VIBES's function amidst varying gastric contents, which may dampen the induced vibrations to varying degrees. The location of the VIBES pill is not controllable and likely to shift during a meal. This does not impede its function in the stomach, as receptors in the antrum and cardia are predominantly stretch sensitive (*10*). In addition, VIBES can consistently create a stretch response as gastric tension receptors are slowly adapting as compared to mucosal mechanoreceptors, which fire in short bursts as seen in our and prior studies (*11*). The large animal study demonstrates that over a 2-week period and despite variations in the animal's routine, sleeping patterns, and activity, food intake was consistently diminished when treated with VIBES. The response to the intervention also reinforces earlier studies highlighting the predominant influence of gastric distension in the satiety response. Use of the VIBES resulted in no observable distress or negative side effects and normal passage in more than 20 trials in a large animal model, supporting the preclinical safety in a relevant animal model.

Weight loss studies are most commonly conducted in rodent models, given practical constraints of cost and resources, similarity in physiology, and ease of dietary-induced disease models (*42*). Our choice of animal model was the swine, given the need for human-sized anatomy accommodating the geometric dimensions of the VIBES pill. However, weight loss is difficult to measure meaningfully in the young and growing swine species that are used for laboratory research (*43*). Future work will devise miniaturized VIBES devices apt for smaller animal model studies, be performed in a dog model, which has a stomach geometry more similar to the human, and use metrics like scintigraphy of liquid and solid meals, and gastric accommodation to further characterize the physiological effects. Future studies should delve deeper into potential correlations between the observed increases in inactive behaviors and mechanisms of postprandial somnolence using longer studies. In addition, the effect of the VIBES traversing through the small intestine on nutrient absorption, microbiome changes, cytokine influences, and overall function of the GI tract should be thoroughly investigated. Mechanisms to externally control the pill's activation or deactivation could also be developed to increase safety and convenience.

Following further safety validations, clinical translation could facilitate a paradigm shift in potential therapeutic options for diseases such as obesity, polyphagia, and Prader-Willi syndrome in which late onset of satiety yields excessive overeating and subsequent metabolic, cardiac, and endocrine comorbidities. Future studies should compare the efficacy and side effects of the VIBES pill to other FDA-approved drugs for weight control or emerging approaches like electrical stimulation (*44*). The metabolic response triggered by VIBES could also be leveraged to treat diseases of insulin insufficiency or dysregulation such as in type II diabetes. Further, the VIBES' ability to increase the motility rate should be investigated. Optogenetic activation of mechanoreceptors such as IGLEs could be another way in in which we could modulate the receptor physiology in the stomach. Overall, this study lays the foundation for a vibratory modality of vagal stimulation, acting through gastric mechanoreceptors, to induce an illusory sense of satiety, decrease food intake, and limit the rate of weight gain, paving the way for a treatment for obesity.

METHODS

Design and modeling of the VIBES

The VIBES was modeled in SOLIDWORKS. The pill was designed to be the same dimensions as a triple zero capsule. The pill was designed with three sections that press-fit onto each other to create a tight seal, in which the two sections housing the electronics are completely sealed off from the third section, where gastric fluid is allowed to enter the capsule to dissolve the glucose layer and activate the pill. The vibration is achieved via an offset weight on the shaft of

a DC motor. Because of its fabrication by 3D printing, a wall thickness of 0.6 mm was used in concordance with the capabilities of the Stratasys printer for the pill capsule's thickness. The 1.55-V, 80-mA·hour silver oxide battery (DigiKey) was used for its high capacity-to-size ratio and prior approvals for use in ingestible biomedical devices, such as ingestible endoscopy capsules (*45*). A spring-loaded mechanism using a pogo pin (DigiKey) was designed to activate the pill once it reached the stomach. The pill was designed to allow gastric fluid to enter through the pill cap and dissolve a glucose membrane separating the negative lead of the battery from the negative lead of the motor.

Fabrication of VIBES
The outer capsule of the VIBES was 3D printed using the Stratasys VeroClear photopolymer due to its strength, transparency, and chemical resistance, making it the ideal material for prototyping. The pill is split into three sections: the motor cap, the central body, and the pill cap. To assemble, the vibrating motor was first pressed into the motor cap. A copper pad is soldered on the positive lead and affixed to the bottom of the motor. The spring loaded pogo pin was then pressed into the central body, and the battery was inserted such that the negative terminal was in contact with the pogo pin. The central body assembly was then pressed onto the motor cap assembly such that the positive terminal of the battery contacts the positive copper pad on the motor. The negative lead of the motor was stretched through the central body to the pill capsule. The negative lead was soldered onto a conductive pad in the top section that, when fully assembled, closed the circuit by contacting the pogo pin. A gelatin membrane was also placed between the negative lead pad and the pogo pin that is designed to dissolve once the pill is immersed or in contact with gastric fluid, allowing it only to be activated when it reaches the stomach (fig. S10). This gelatin is able to dissolve in pH ranging between 1 and 6.8, enabling function even in patients with gastric fluid of varying pH (*46*).

Pill characterization (vibration time test and chemical resistance test)
The pill's vibration time was characterized using 80-mA·hour silver oxide batteries by measuring the amount of time it took for the pill to stop vibrating on a full battery. Chemical degradation of the pill is a concern because of contact with acidic stomach fluid. We expect residence in the stomach for no more than 4 hours in a healthy animal with normal motility. Using a safety factor of 6, the chemical resistance of the pill was evaluated via a 24-hour submersion in simulated gastric fluid (pH 1.2; Ricca, CAS #7732-18-5) and 10-day immersion in simulated intestinal fluid (pH 7; Ricca CAS #TS-7109-32). After this incubation period, the pill was visually evaluated, mechanically inspected for defects, abrasion, or weakening. Furthermore, the electronic components were activated to determine whether the pill could be activated.

Thermal risk testing
To evaluate the thermal safety of the pill, we measured heat output in water and air using the FLIR A65SC Test Kit and a thermal black body (Dahua Technology JQ-D70Z) as a calibration standard. The pill was vibrated at 25, 80, 95, 110, 200, 250, and 300 Hz for 30 min each in a beaker with 20 ml of saline to determine the average, minimum, and maximum temperatures achieved at each frequency in the surrounding fluid.

In vivo experiments
All animal experiments were conducted in accordance with protocols approved by the Committee on Animal Care at the Massachusetts Institute of Technology (MIT). Studies were performed in a swine model [50- to 80-kg Yorkshire pigs ranging between 4 and 6 months of age from Cummings Veterinary School at Tufts University (Grafton, MA)]. The swine model was chosen because its gastric anatomy is similar to that of humans and has been widely used in the evaluation of biomedical GI devices (*32*). Unless otherwise noted, animals were anesthetized using an intramuscular injection of dexmedetomidine (0.03 mg/kg) and midazolam (0.25 mg/kg), intubated, and maintained on 2 to 3% isoflurane in oxygen. For mechanistic studies in which the VIBES pill was placed directly in the stomach, an upper GI endoscopy was performed to place an overtube to the stomach. Then, the VIBES pill was delivered to the stomach directly.

Animals in the experimental and control groups underwent the same experimental manipulation (anesthesia, endoscopy, blood collection, x-ray, feeding, handling, and electrophysiological procedures). In the experimental group, a VIBES pill performing stimulation was used, with manual confirmation of a working pill in each trial. In the control groups, a sham pill without any functionality was used, unless otherwise noted. A blinded endoscopist assessed the stomach for any morphological changes, signs of damage, or adverse effects in both control (n = 6) animals and those treated with the VIBES pill (n = 6).

Passage study
To assess potential effects on motility, animals were either fed a sham pill (n = 3 swine) or the VIBES pill (n = 3 swine) along with a set of small radio-opaque barium pellets. In the experimental group, the VIBES pill was hidden in a piece of banana and hand fed to the animals. Care was taken to ensure that the animals did not bite or chew the pill to cause breakage before swallowing. All animals were monitored by x-ray imaging every other day until the pill was passed. Any changes in behavior, eating, or indications of pain or distress were monitored and recorded using a video camera placed near the cage (MIPC systems).

Electrophysiology
In terminal procedures, following laparotomy and dissection, the celiac branch of the vagus nerve, running alongside the left gastro-omental arteries, was identified. A set of 10 to 12 30-gauge fine wire electrodes were inserted into the celiac branch of the vagus nerve using 26 gauge needles. The stomach was inflated to differing degrees using the endoscopic air inflation/suction channel by monitoring the volume and flow rate of the air. Electrical signals were recorded using the Intan Technologies Recording and Stimulation System at 30 kHz. Twenty minutes of baseline signals were recorded. Then, the stomach was inflated to differing degrees using the endoscopic air inflation/suction channel. The volume of the stomach was first determined by fully inflating the stomach, observing the separation of rugae and measuring the volume of air used. 30, 60, and 90% of these volumes and separation levels were used for a graded study of the neuronal spiking physiology. Then, another 20 min of baseline was recorded. The VIBES pill was then introduced into the stomach, and electrical signals were recorded in a similar manner. Vagotomies were conducted at the cervical level, proximal to the placement of recording electrodes.

Data were exported and processed in MATLAB. Data were bandpass– and notch-filtered (60 Hz) to remove low-frequency motion artifacts, breathing artifacts, and white noise. A cutoff frequency of 1000 was used with a fifth-order Butterworth high-pass filter. Signals were rectified to assess amplitudes. The signals generated in each group were averaged, and fast Fourier transforms were performed to compare the spectral properties between groups.

Statistical analysis of afferent electroneurographic signals

To determine the statistical significance of the computed spectral similarity measures, a two-sample *t* test with a significance level (alpha) set at 0.05. The magnitude of the FFT signals was used as the input for the *t* test. Furthermore, cross-spectral coherence was calculated as follows

$$\text{coherence} = \text{abs}\{\text{sum}[\text{conjugate}(X1).*X2]\}/\text{sqrt}\{\text{sum}[\text{abs}(X1).^{2}]^{*}\ \text{sum}[\text{abs}(X2).^{2}]\}$$

An alpha of 0.05 was used to assess significance.

Serotonin screening

A 6 × 4 well Franz cell apparatus was used to test mucosal serotonin release in ex vivo tissue from swine. VIBES treatment was applied for 20 min, and luminal secretions were sampled by washes of 200 μL and compared to that of untreated control tissue. Six independent samples were collected at three time points. A serotonin enzyme-linked immunosorbent assay (ELISA; Enzo Bio Sciences) with fluorescent readout was used to quantify the secretions.

Gastric fluid pH testing

A 6 × 4 well Franz cell apparatus was used to test the effect of the VIBES pill on the pH of gastric fluid. Gastric fluid collected from swine was placed into the donor and recipient wells of the apparatus sandwiching tissue from swine. VIBES treatment was applied for 20 min in the experimental samples. The pH of the fluid on the luminal side was measured using a Mettler-Toledo pH meter in control and experimental samples. Six independent samples were collected from each group.

Metabolic panel

A metabolic hormone panel (Eve Technologies) was performed on venous blood collected from an ear vein catheter in control (n = 6) and experimental (n = 6) animals. Sham pills were administered to the control group, while VIBES pills were administered to the experimental group. Blood was collected 30 min before stimulation and every 30 min for 2 hours in awake and freely moving animals (n = 6). Collection vials spray coated with EDTA were inverted five times each after filling with blood samples. Blood was treated with a protease inhibitor cocktail (S8830, Sigma-Aldrich) within 10 min of collection and centrifuged for 15 min at 4°C at 4000 rpm. Blood analyzed for GLP-1 was treated with DPPIV inhibitor (Sigma-Aldrich) within 2 min of collection and centrifuged for 15 min at 4°C at 4000 rpm. ELISA results were normalized to the baseline values. Control samples were run with every assay run to ensure that all assay signals of a particular lot fall within an acceptable range. We used a two-tailed heteroscedastic *t* test to compare the average value of the hormone after stimulation between the control and VIBES groups at an alpha of 0.05. Glucose was measured using a handheld glucometer with blood drawn from a venous catheter placed in the ear.

Two-week feeding study

Repeated oral administration of an electronic pill in a swine model is challenging, as it is hard to prevent biting or chewing. Furthermore, the manual handling can become a source of distress for the animals. Thus, we loosely tethered our device in the gastric cavity using a percutaneous gastrostomy (PEG) tube. We externalized the battery source to enable daily operation during treatment times. The tethered VIBES pill (fig. S11 and movie S3) was inserted through a 28-gauge PEG tube (AVANOS) that was placed with endoscopic assistance. Daily stimulation was then performed for 30 min, 20 to 30 min before mealtimes at 7:30 a.m. and 3:30 p.m. The animal was fed LabDiet mini-pig grower pellets (5081) at 7:30 a.m. and 3:30 p.m. and a snack of five apples between 11 a.m. and 12 p.m. The mass of the food provided and leftover in the hopper (or anything spilled nearby) after 30 min were measured. Videography of stimulation and feeding periods was analyzed to surveil for any changes in behavior, appetite, and side effects such as nausea, vomiting, lethargy, etc. The animal's body weight was measured at least twice per week, and fresh fecal samples were collected twice a week after stimulation.

Image-based behavioral study

To capture trends and insights of the behaviors exhibited by the subjects in the control and treated conditions, we developed a deep learning architecture. Prioritizing interpretability, we drew on Lehner's ethological model of innate behavior to formalize the problem of classifying behavior and chains of behavior (fig. S12) (*47*). Vibration is the stimulation being given by the VIBES, the releaser, to the animal before food ingestion. VIBES may then influence eating, drinking, and activity levels. Within operant condition behavior theory, the consequences of satiation have neither positive nor aversive reinforcing value; the feedback mechanism from consequences is time dependent. Initially, feeding is a positively reinforcing stimulation; however, the value of the reinforcer decreases over time until the animal reaches satiety. If the animal continues to eat, then feeding becomes a negatively reinforcing stimulation, increasing in negative value as the animal continues to feed. The next behavioral act in the continuous stream of behavior can be elicited by the consequence of the behavior that precedes it.

To classify the behaviors exhibited by the subjects and track differences between conditions over time, we collected audio and video data of the pigs in the animal facilities using 1080p HD IP (network) cameras with night vision and audio mounted in the corner at the top of the pen. Animals were held in woodchip embedded pens (5 inches by 5 inches) with toys, water spigot, and food supply fixed to the walls. We collected continuous 1920 × 1080 resolution RGB video data at a rate of 15 FPS and audio data at 16,000 Hz. We collected data from four pigs in two pens for six noncontinuous days for a total of 94 hours.

For the analysis, we use a three-stage pipeline, as shown in fig. S13 below. In the first stage, markerless pose estimation was performed on raw video data with 2D pose integration. We trained a model to capture time-series estimations for the positional coordinates of 22 different body parts and 10 objects in the environment. This incorporated a deep convolutional neural network that has shown inter- and intraspecies transfer learning capabilities. We

trained the model on a dataset consisting of 420 frames over 12 hours of daytime video. Three independent video coders annotated 420 image frames were labeled spanning a variety of perspectives and colors to help increase performance. The set of image frames were randomly sampled while preserving the representation of the distinct view angles, variety of illumination levels, and different resolutions and video qualities. To verify label consistency, we generated a 10-pixel radius around the original labels and calculated Cronbach's alpha to measure the inter-rater reliability between video coders, resulting in a score of 0.837 and demonstrating a high internal consistency of labels. We labeled 22 different key points composed of relevant pig body parts and objects in the environment (table S2).

For body part tracking we used DeepLabCut (version 2.2.1.1) (*18*, *48*, *49*). Specifically, we labeled 420 frames taken from 21 videos/animals [then, 95% was used for training; we used a ResNet-50–based neural network with default parameters for 400,000 training iterations; we validated with one shuffle and found that the test error was 6.34 pixels, train: 9.58 pixels (image size was 1920 by 1080)]. We then used a P-cutoff of 0.6 to condition the X,Y coordinates for future analysis. This network was then used to analyze videos from similar experimental settings.

For the second stage, we used the positional coordinate time series output and derived features, such as angles between the water spigot, head, and tail, from the DeepLabCut model to estimate the behaviors exhibited by the pigs. A single day's worth (24 hours) of video data from each participant was coded using the ethograms below. The primary coder was blind to experimental conditions until after analysis. Secondary coders were not blind to the experimental conditions. We report strong inter-rater reliability between coders ($\kappa = 0.92$). The data generated from these labels was used to train a two-layer long short-term memory (LSTM) network with 100 fully connected, hidden units and estimate the pig's behaviors during a window of time. This model was trained for 600 epochs with a learning rate of 0.001 and resulted in a max (early stopping) weighted F1 score of 93.5%.

The output of the LSTM network classified 4 general categories of behaviors, such as sleeping and eating from the feeder (table S3). Classes are imbalanced within the categories with as many as 284,004 data points for "resting" and as few as 2103 for "being fed Pedialyte." To quantify the feature detector's performance, we use fivefold cross-validation across each hour of image data.

The k-fold cross-validation included (i) the division of the dataset into randomly sampled, independent k-folds without replacement, (ii) $K - 1$ folds used for model training, and the remaining used for performance evaluation (iii) repetition of prior step k times to obtain k number of performance estimates for each iteration, and (iv) a mean of k number of performance estimates. We then evaluated the performance of LSTMs on test images across all generated features.

To capture model performance, we provide the confusion matrix of the behaviors and the model's accuracy over time. On average, our model classifies the four different general categories of behavior with 94% accuracy (figs. S15 and S16).

The data output from DeepLabCut was a time-series positional coordinate dataset paired with activity labels and a series of images. From here, we used LSTMs to infer latents and states. We use the latents and stages to calculate features such as time, frequency, duration, and trends of behaviors across days in the third stage.

Histology

Following euthanasia, stomach sections were carefully harvested from animals in the control and experimental groups. Tissue samples were fixed in 4% paraformaldehyde for 24 hours. They were then washed in phosphate-buffered saline three times for 15 min each and stored in 70% ethanol. They were then paraffin-processed, embedded, and then sectioned. Tissues were stained with (i) hematoxylin and eosin to assess morphology and surveil for adverse side effects related to the intervention.

Statistical analyses

Quantitative data are reported as means (±SD) or as a range when appropriate. The normality of the distributions was checked by the Shapiro-Wilk test. Comparative analyses were performed using Student's heteroscedastic two-tailed t test, unless otherwise noted. $P < 0.05$ was considered significant.

Supplementary Materials

This PDF file includes:
Figs. S1 to S16
Note S1
Table S1 to S3
Legends for movies S1 to S3

Other Supplementary Material for this manuscript includes the following:
Movie S1 to S3

REFERENCES AND NOTES

1. CDC, *Obesity is a Common, Serious, and Costly Disease* (CDC, 2022); www.cdc.gov/obesity/data/adult.html.
2. NIDDK, Overweight & Obesity Statistics | NIDDK, www.niddk.nih.gov/health-information/health-statistics/overweight-obesity.
3. F. L. Greenway, L. J. Aronne, A. Raben, A. Astrup, C. M. Apovian, J. O. Hill, L. M. Kaplan, K. Fujioka, E. Matejkova, S. Svacina, L. Luzi, L. Gnessi, S. Navas-Carretero, J. Alfredo Martinez, C. D. Still, A. Sannino, C. Saponaro, C. Demitri, L. E. Urban, H. Leider, E. Chiquette, E. S. Ron, Y. Zohar, H. M. Heshmati, A randomized, double-blind, placebo-controlled study of Gelesis100: A novel nonsystemic oral hydrogel for weight loss. *Obes. Silver Spring Md* **27**, 205–216 (2019).
4. GBD 2015 Obesity Collaborators, A. Afshin, M. H. Forouzanfar, M. B. Reitsma, P. Sur, K. Estep, A. Lee, L. Marczak, A. H. Mokdad, M. Moradi-Lakeh, M. Naghavi, J. S. Salama, T. Vos, K. H. Abate, C. Abbafati, M. B. Ahmed, Z. Al-Aly, A. Alkerwi, R. Al-Raddadi, A. T. Amare, A. Amberbir, A. K. Amegah, E. Amini, S. M. Amrock, R. M. Anjana, J. Ärnlöv, H. Asayesh, A. Banerjee, A. Barac, E. Baye, D. A. Bennett, A. S. Beyene, S. Biadgilign, S. Biryukov, E. Bjertness, D. J. Boneya, I. Campos-Nonato, J. J. Carrero, P. Cecilio, K. Cercy, L. G. Ciobanu, L. Cornaby, S. A. Damtew, L. Dandona, R. Dandona, S. D. Dharmaratne, B. B. Duncan, B. Eshrati, A. Esteghamati, V. L. Feigin, J. C. Fernandes, T. Fürst, T. T. Gebrehiwot, A. Gold, P. N. Gona, A. Goto, T. D. Habtewold, K. T. Hadush, N. Hafezi-Nejad, S. I. Hay, M. Horino, F. Islami, R. Kamal, A. Kasaeian, S. V. Katikireddi, A. P. Kengne, C. N. Kesavachandran, Y. S. Khader, Y.-H. Khang, J. Khubchandani, D. Kim, Y. J. Kim, Y. Kinfu, S. Kosen, T. Ku, B. K. Defo, G. A. Kumar, H. J. Larson, M. Leinsalu, X. Liang, S. S. Lim, P. Liu, A. D. Lopez, R. Lozano, A. Majeed, R. Malekzadeh, D. C. Malta, M. Mazidi, C. McAlinden, S. T. McGarvey, D. T. Mengistu, G. A. Mensah, G. B. M. Mensink, H. B. Mezgebe, E. M. Mirrakhimov, U. O. Mueller, J. J. Noubiap, C. M. Obermeyer, F. A. Ogbo, M. O. Owolabi, G. C. Patton, F. Pourmalek, M. Qorbani, A. Rafay, R. K. Rai, C. L. Ranabhat, N. Reinig, S. Safiri, J. A. Salomon, J. R. Sanabria, I. S. Santos, B. Sartorius, M. Sawhney, J. Schmidhuber, A. E. Schutte, M. I. Schmidt, S. G. Sepanlou, M. Shamsizadeh, S. Sheikhbahaei, M.-J. Shin, R. Shiri, I. Shiue, H. S. Roba, D. A. S. Silva, J. I. Silverberg, J. A. Singh, S. Stranges, S. Swaminathan, R. Tabarés-Seisdedos, F. Tadese, B. A. Tedla, B. S. Tegegne, A. S. Terkawi, J. S. Thakur, M. Tonelli, R. Topor-Madry, S. Tyrovolas, K. N. Ukwaja, O. A. Uthman, M. Vaezghasemi, T. Vasankari, V. V. Vlassov, S. E. Vollset, E. Weiderpass, A. Werdecker, J. Wesana, R. Westerman, Y. Yano, N. Yonemoto, G. Yonga, Z. Zaidi, Z. M. Zenebe, B. Zipkin, C. J. L. Murray, Health effects of overweight and obesity in 195 countries over 25 years. *N. Engl. J. Med.* **377**, 13–27 (2017).

5. D. Boyers, L. Retat, E. Jacobsen, A. Avenell, P. Aveyard, E. Corbould, A. Jaccard, D. Cooper, C. Robertson, M. Aceves-Martins, B. Xu, Z. Skea, M. de Bruin; the REBALANCE team, Cost-effectiveness of bariatric surgery and non-surgical weight management programmes for adults with severe obesity: a decision analysis model. *Int. J. Obes.* **45**, 2179–2190 (2021).
6. B. N. Lauren, F. Lim, A. Krikhely, E. M. Taveras, J. A. Woo Baidal, B. K. Bellows, C. Hur, Estimated cost-effectiveness of medical therapy, sleeve gastrectomy, and gastric bypass in patients with severe obesity and type 2 diabetes. *JAMA Netw. Open* **5**, e2148317 (2022).
7. B. Doble, S. Wordsworth, C. A. Rogers, R. Welbourn, J. Byrne, J. M. Blazeby; By-Band-Sleeve Trial Management Group, What are the real procedural costs of bariatric surgery? A systematic literature review of published cost analyses. *Obes. Surg.* **27**, 2179–2192 (2017).
8. P. Janssen, P. V. Berghe, S. Verschueren, A. Lehmann, I. Depoortere, J. Tack, Review article: The role of gastric motility in the control of food intake. *Aliment. Pharmacol. Ther.* **33**, 880–894 (2011).
9. H.-R. Berthoud, Vagal and hormonal gut–brain communication: From satiation to satisfaction. *Neurogastroenterol. Motil.* **20**, 64–72 (2008).
10. V. P. Zagorodnyuk, B. N. Chen, S. J. Brookes, Intraganglionic laminar endings are mechanotransduction sites of vagal tension receptors in the guinea-pig stomach. *J. Physiol.* **534**, 255–268 (2001).
11. A. E. Willing, H. R. Berthoud, Gastric distension-induced c-fos expression in catecholaminergic neurons of rat dorsal vagal complex. *Am. J. Physiol.* **272**, R59–R67 (1997).
12. G. de Lartigue, Role of the vagus nerve in the development and treatment of diet-induced obesity. *J. Physiol.* **594**, 5791–5815 (2016).
13. M. Camilleri, Peripheral mechanisms in appetite regulation. *Gastroenterology* **148**, 1219–1233 (2015).
14. C. R. Plata-Salamán, Regulation of hunger and satiety in man. *Dig. Dis.* **9**, 253–268 (2004).
15. G. Grabauskas, C. Owyang, Plasticity of vagal afferent signaling in the gut. *Medicina* **53**, 73–84 (2017).
16. S. L. Hargrave, K. P. Kinzig, Repeated gastric distension alters food intake and neuroendocrine profiles in rats. *Physiol. Behav.* **105**, 975–981 (2012).
17. G. J. Schwartz, P. R. McHugh, T. H. Moran, Integration of vagal afferent responses to gastric loads and cholecystokinin in rats. *Am. J. Physiol.* **261**, R64–R69 (1991).
18. C. Mathis, T. H. Moran, G. J. Schwartz, Load-sensitive rat gastric vagal afferents encode volume but not gastric nutrients. *Am. J. Physiol.* **274**, R280–R286 (1998).
19. C. M. Tate, A. Geliebter, Intragastric balloon treatment for obesity: Review of recent studies. *Adv. Ther.* **34**, 1859–1875 (2017).
20. D. Rigaud, N. Trostler, R. Rozen, T. Vallot, M. Apfelbaum, Gastric distension, hunger and energy intake after balloon implantation in severe obesity. *Int. J. Obes. Relat. Metab. Disord.* **19**, 489–495 (1995).
21. F. L. Greenway, Physiological adaptations to weight loss and factors favouring weight regain. *Int. J. Obes.* **39**, 1188–1196 (2015).
22. S. J. Choi, H. S. Choi, Various intragastric balloons under clinical investigation. *Clin. Endosc.* **51**, 407–415 (2018).
23. FDA, UPDATE: Potential Risks with Liquid-filled Intragastric Balloons—Letter to Health Care Providers. *FDA* (2020); www.fda.gov/medical-devices/letters-health-care-providers/fda-alerts-health-care-providers-about-potential-risks-liquid-filled-intragastric-balloons.
24. B. Bonaz, C. Picq, V. Sinniger, J. F. Mayol, D. Clarençon, Vagus nerve stimulation: From epilepsy to the cholinergic anti-inflammatory pathway. *Neurogastroenterol. Motil.* **25**, 208–221 (2013).
25. J. G. Kral, L. Görtz, G. Hermansson, G. S. Wallin, Gastroplasty for obesity: Long-term weight loss improved by vagotomy. *World J. Surg.* **17**, 75–78 (1993).
26. M. Wierdak, E. Korbut, M. Hubalewska-Mazgaj, M. Surmiak, K. Magierowska, D. Wójcik-Grzybek, M. Pędziwiatr, T. Brzozowski, M. Magierowski, Impact of vagotomy on postoperative weight loss, alimentary intake, and enterohormone secretion after bariatric surgery in experimental translational models. *Obes. Surg.* **32**, 1586–1600 (2022).
27. G. D. Miller, Appetite regulation: Hormones, peptides, and neurotransmitters and their role in obesity. *Am. J. Lifestyle Med.* **13**, 586–601 (2017).
28. G. M. Goodwin, D. I. McCloskey, P. B. C. Matthews, Proprioceptive illusions induced by muscle vibration: Contribution by muscle spindles to perception? *Science* **175**, 1382–1384 (1972).
29. S. Heo, J. Lee, D. Wigdor, *in Proceedings of the 32nd Annual ACM Symposium on User Interface Software and Technology, UIST '19* (Association for Computing Machinery, 2019), pp. 803–813.
30. Muscle Vibration-Induced Illusions: Review of Contributing Factors, Taxonomy of Illusions and User's Guide in: Multisensory Research Volume 30 Issue 1 (2017); https://brill-com.ezp-prod1.hul.harvard.edu/view/journals/msr/30/1/article-p25_2.xml.
31. G. Carnaby-Mann, M. Crary, Pill swallowing by adults with dysphagia. *Arch. Otolaryngol. Neck Surg.* **131**, 970–975 (2005).
32. K. Stamatopoulos, C. O'Farrell, M. Simmons, H. Batchelor, In vivo models to evaluate ingestible devices: Present status and current trends. *Adv. Drug Deliv. Rev.* **177**, 113915 (2021).
33. L. A. Blackshaw, D. Grundy, Effects of cholecystokinin (CCK-8) on two classes of gastroduodenal vagal afferent fibre. *J. Auton. Nerv. Syst.* **31**, 191–201 (1990).
34. A. E. Foxx-Orenstein, J. F. Kuemmerle, J. R. Grider, Distinct 5-HT receptors mediate the peristaltic reflex induced by mucosal stimuli in human and guinea pig intestine. *Gastroenterology* **111**, 1281–1290 (1996).
35. G. M. Mawe, J. M. Hoffman, Serotonin signaling in the gastrointestinal tract. *Nat. Rev. Gastroenterol. Hepatol.* **10**, 473–486 (2013).
36. J. M. Kellum, F. C. Albuquerque, M. C. Stoner, R. P. Harris, Stroking human jejunal mucosa induces 5-HT release and Cl- secretion via afferent neurons and 5-HT4 receptors. *Am. J. Physiol.* **277**, G515–G520 (1999).
37. T. von Erlach, S. Saxton, Y. Shi, D. Minahan, D. Reker, F. Javid, Y.-A. L. Lee, C. Schoellhammer, T. Esfandiary, C. Cleveland, L. Booth, J. Lin, H. Levy, S. Blackburn, A. Hayward, R. Langer, G. Traverso, Robotically handled whole-tissue culture system for the screening of oral drug formulations. *Nat. Biomed. Eng.* **4**, 544–559 (2020).
38. J. Maselyne, W. Saeys, A. Van Nuffel, Review: Quantifying animal feeding behaviour with a focus on pigs. *Physiol. Behav.* **138**, 37–51 (2015).
39. J. A. de Leeuw, J. E. Bolhuis, G. Bosch, W. J. J. Gerrits, Effects of dietary fibre on behaviour and satiety in pigs. *Proc. Nutr. Soc.* **67**, 334–342 (2008).
40. J. A. de Leeuw, A. W. Jongbloed, M. W. A. Verstegen, Dietary fiber stabilizes blood glucose and insulin levels and reduces physical activity in sows (Sus scrofa). *J. Nutr.* **134**, 1481–1486 (2004).
41. K. N. Browning, Role of central vagal 5-HT3 receptors in gastrointestinal physiology and pathophysiology. *Front. Neurosci.* **9**, 413 (2015).
42. S. P. Vickers, H. C. Jackson, S. C. Cheetham, The utility of animal models to evaluate novel anti-obesity agents. *Br. J. Pharmacol.* **164**, 1248–1262 (2011).
43. J. M. Bell, A study of rates of growth of yorkshire, lacombe, landrace, and crossbred pigs from birth to 200 lb. *Can. J. Anim. Sci.* **44**, 315–319 (1964).
44. K. B. Ramadi, J. C. McRae, G. Selsing, A. Su, R. Fernandes, M. Hickling, B. Rios, S. Babaee, S. Min, D. Gwynne, N. Z. Jia, A. Aragon, K. Ishida, J. Kuosmanen, J. Jenkins, A. Hayward, K. Kamrin, G. Traverso, Bioinspired, ingestible electroceutical capsules for hunger-regulating hormone modulation. *Sci. Robot.* **8**, eade9676 (2023).
45. S.-Y. Yang, V. Sencadas, S. S. You, N. Z.-X. Jia, S. S. Srinivasan, H.-W. Huang, A. E. Ahmed, J. Y. Liang, G. Traverso, Powering implantable and ingestible electronics. *Adv. Funct. Mater.* **31**, 2009289 (2021).
46. V. Gray, E. Cole, J. Toma, L. Ghidorsi, J.-H. Guo, J.-H. Han, F. Han, C. Hosty, J. Kochling, J. Kraemer, T. Langdon, S. Leinbach, G. Martin, S. Meyerhoffer, R. Moreton, K. Raghavan, E. Shneyvas, J. Suggett, S. Tindal, M. Marques, Use of enzymes in the dissolution testing of gelatin capsules and gelatin-coated tablets-revisions to dissolution < 711 > and disintegration and dissolution of dietary supplements < 2040 >. *Dissolution Technol.* **21**, 6–18 (2014).
47. P. N. Lehner, *Handbook of Ethological Methods* (Cambridge Univ. Press, ed. 2, 1998).
48. T. Nath, A. Mathis, A. C. Chen, A. Patel, M. Bethge, M. W. Mathis, Using DeepLabCut for 3D markerless pose estimation across species and behaviors. *Nat. Protoc.* **14**, 2152–2176 (2019).
49. A. Mathis, P. Mamidanna, K. M. Cury, T. Abe, V. N. Murthy, M. W. Mathis, M. Bethge, DeepLabCut: Markerless pose estimation of user-defined body parts with deep learning. *Nat. Neurosci.* **21**, 1281–1289 (2018).

Acknowledgments: We are grateful for constructive feedback from R. Langer. Original artwork and illustrations by V. E. Fulford, Alar Illustration. We thank H. Sun for assistance with labeling data. **Funding:** This work was supported in part by grants from the NIH (EB-000244), Novo Nordisk, and Department of Mechanical Engineering, MIT. S.S. was supported by a Schmidt Science Fellowship. C.B. was supported by the NSF under grant #2030859 to the Computing Research Association for the CIFellows Project. **Author contributions:** Conceptualization: S.S.S. Methodology: A.A., A.H., S.S.S., C.B., and G.T. Investigation: S.S.S., A.A., A.H., C.B., J.K., K.I., J.J., S.L., and W.A.M.M. Supervision: A.H., N.F., and G.T. Writing (original draft): S.S., A.A., A.H., and C.B. Writing (review and editing): All authors. **Competing interests:** S.S., A.A., and G.T. are co-inventors on a patent application (application filed by the Massachusetts Institute of Technology, no. 63/319,620) describing the developments presented here (application no. 63/319,620). G.T. reports receiving consulting fees from Novo Nordisk. Complete details of all relationships for profit and not for profit for G.T. can be found at the following link: https://dropbox.com/sh/szi7vnr4a2ajb56/AABs5N5i0q9AfT1IqIJAE-T5a?dl=0. All authors have submitted an invention disclosure to MIT. All authors declare that they have no other competing interests. **Data and materials availability:** All data needed to evaluate the conclusions in the paper are present in the paper and/or the Supplementary Materials.

Submitted 19 June 2023
Accepted 21 November 2023
Published 22 December 2023
10.1126/sciadv.adj3003
