# Supplementary material for: A vibrating ingestible bioelectronic stimulator modulates gastric stretch receptors for illusory satiety
Source: Sci Adv. 2023 Dec 22;9(51):eadj3003. doi: 10.1126/sciadv.adj3003 (PMC10745699; doi:10.1126/sciadv.adj3003)
Supplement: Supplementary file 2 — Figs. S1 to S16 Note S1 Table S1 to S3 Legends for movies S1 to S3 [file sciadv.adj3003_sm.pdf]

Science Advances
AAAS

Supplementary Materials for

A vibrating ingestible bioelectronic stimulator modulates gastric stretch receptors for illusory satiety

Shriya S. Srinivasan *et al.*

Corresponding author: Giovanni Traverso, cgt20@mit.edu, ctraverso@bwh.harvard.edu;
Shriya S. Srinivasan, shriya_srinivasan@fas.harvard.edu

Sci. Adv. **9**, eadj3003 (2023)
DOI: 10.1126/sciadv.adj3003

The PDF file includes:

Figs. S1 to S16
Note S1
Tables S1 to S3
Legends for movies S1 to S3

Other Supplementary Material for this manuscript includes the following:

Movies S1 to S3

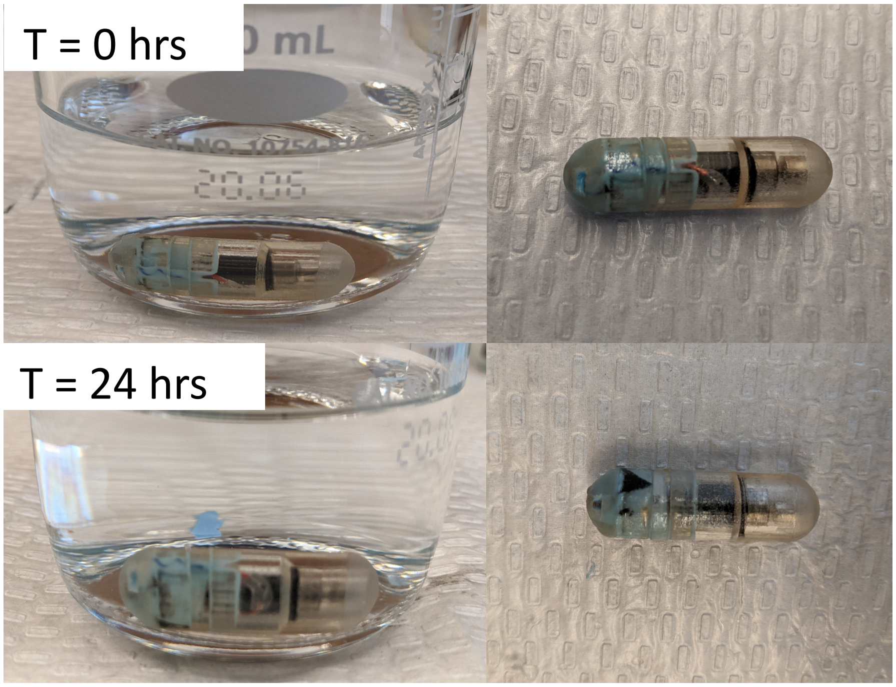

Supplemental Figure 1. Chemical Resistance Test. Submersion in in Simulated Gastric Fluid did not erode the pills surface or damage any internal hardware (bottom) as compared to its pre-submersion state (top). Following 24 hours of submersion, the pill was able to be activated and functioned normally.

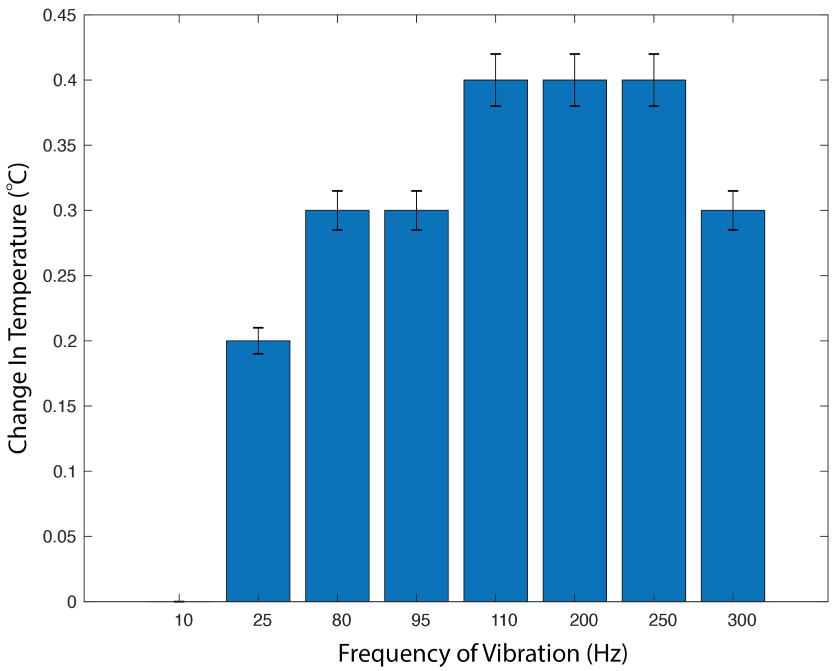

Supplemental Figure 2. Thermal Testing. The VIBES was operated at various frequencies for 30 minutes in 20mL of saline. Change in temperature from baseline was assessed using thermal imaging of the fluid. In all cases, there was less than a 0.5°C increase in the surrounding fluid, indicating that the VIBES does not pose any thermal risk to tissue.

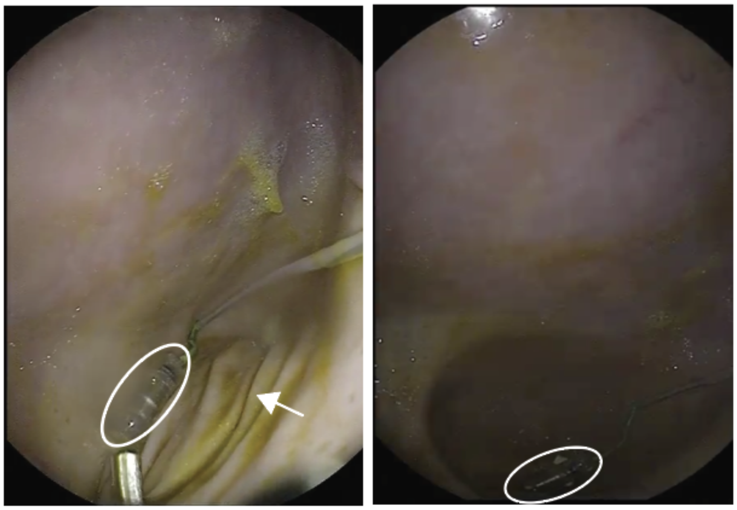

Supplemental Figure 3. Insufflation of the stomach to 30% (left) and 90% (right) can be visualized by the presence of lack of rugae in the stomach (white arrow). Circled is the VIBES pill making mucosal contact.

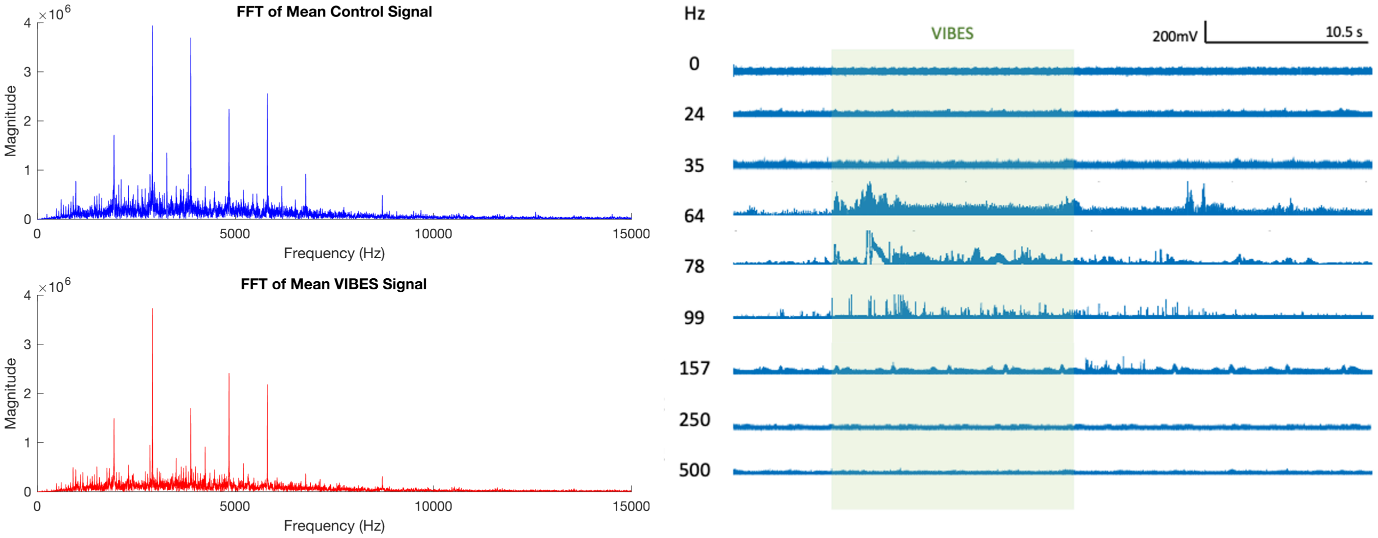

Supplemental Figure 4. Vagal Signaling in response to VIBES. (Left) Comparison of the fast Fourier transform (FFT) of the averaged afferent ENG signals generated on the vagal nerve in the control group (top, blue) and VIBES group (bottom, red), demonstrating highly similar frequency spectra. The highest peak for both signals occurs at 2904 Hz with a magnitude of 3,728,010 in the VIBES group and 3,938,510 in the control group. (Right) Afferent ENG in response to VIBES stimulation at frequencies between 24 – 500 Hz.

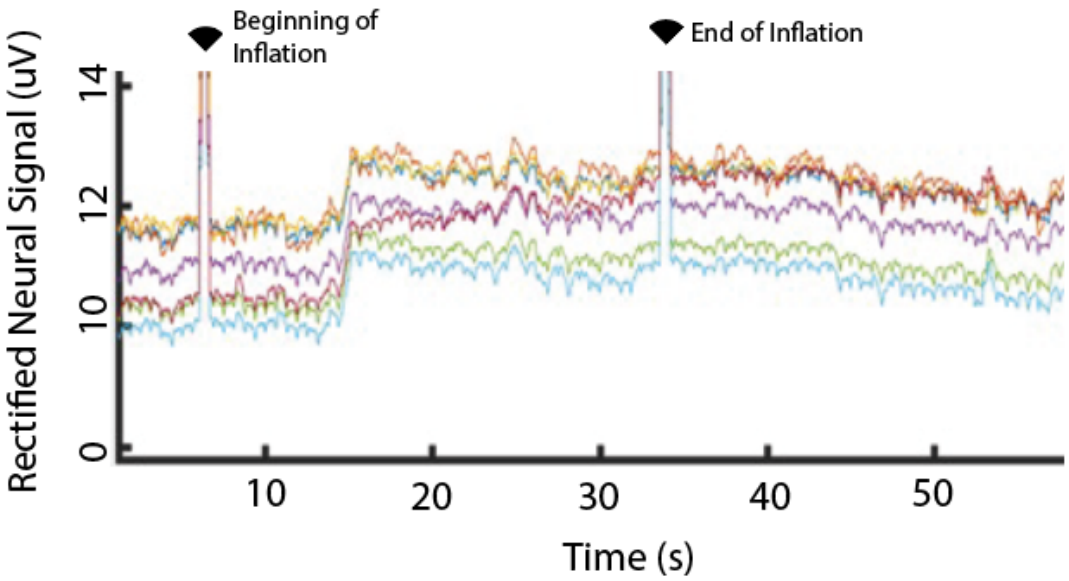

Supplemental Figure 5. Rectified electroneurography demonstrates a sharp increase in spiking 0-12 seconds following the beginning of inflation. In this representative trial, the gastric cavity was insufflated to 90% of its full volume, requiring 38 seconds of insufflation.

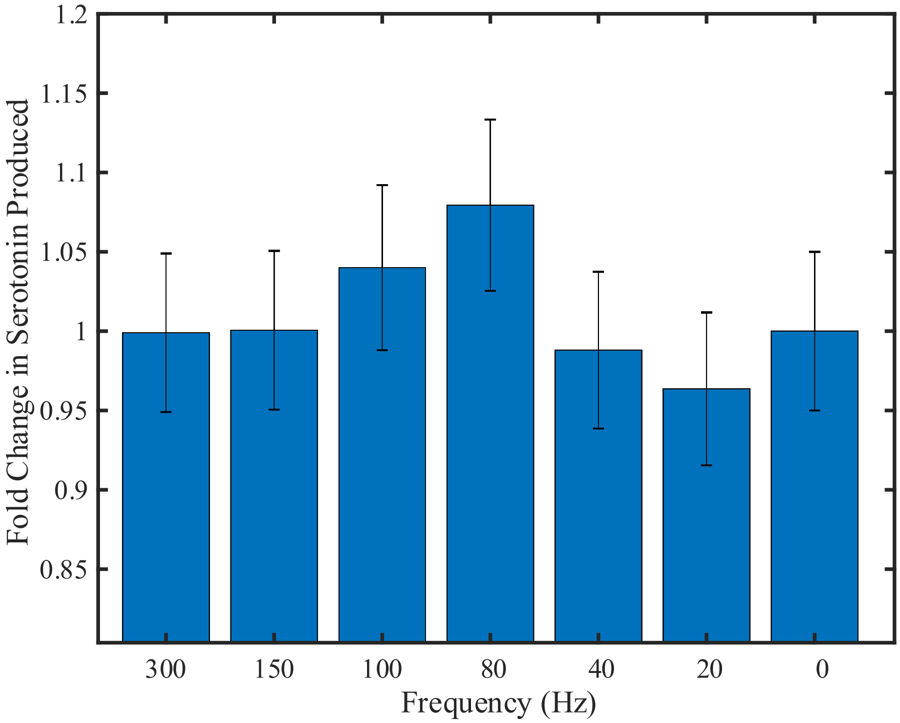

Supplementary Figure 6. Serotonin release on the luminal surface of gastric tissue (n =12 trials using tissue samples from n = 2 swine) was assayed in response to VIBES at varying frequencies.

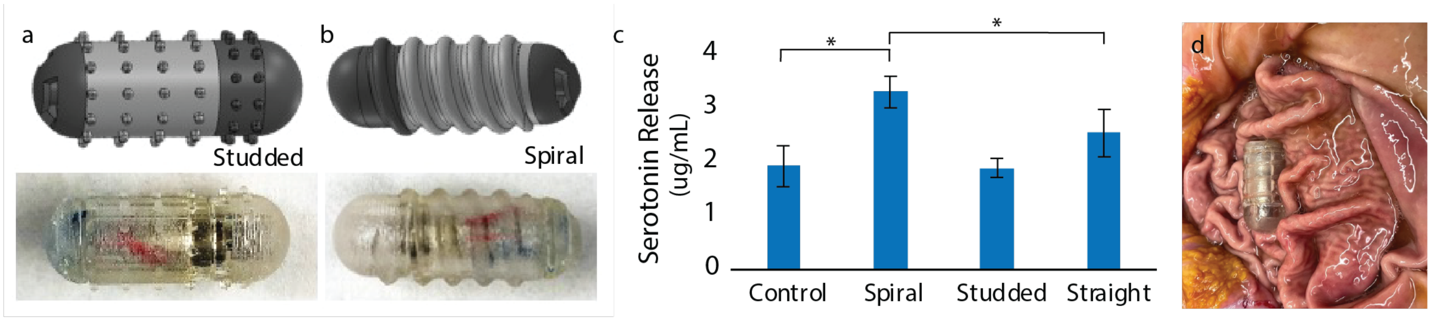

Supplemental Figure 7. Optimizing Surface Geometry for Mucosal Stroking. Surface features of studs (a) and spirals (b) were incorporated to stroke microvilli and rugae during VIBES pill rotation. C) The effect of surface geometry on serotonin release indicates significantly higher serotonin release levels ($p < 0.05$, Student's two tailed t-test) for a spiral design as compared to a straight surface geometry or the control condition, where no VIBES was used. D) Representative image of the spiral VIBES pill seated amongst gastric ruggae.

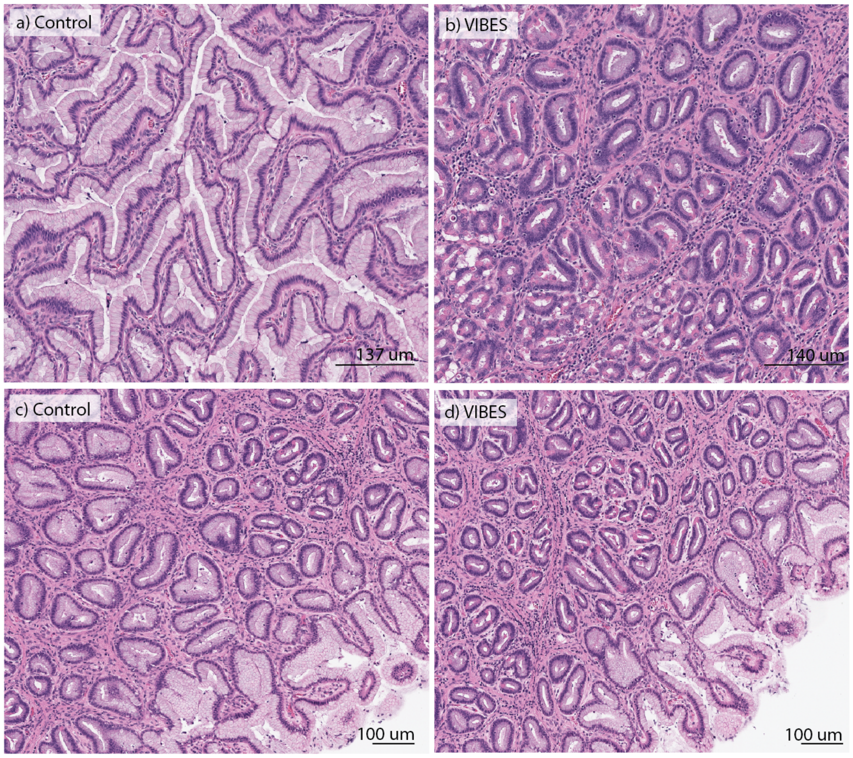

Supplemental Figure 8. Histological analysis of gastric samples. Representative tissue cross sections stained with hematoxylin and eosin from a,c) control untreated animals and b,d) those treated with the VIBES pill. Tissue treated with VIBES shows no marked inflammation, irritation, or morphological changes.

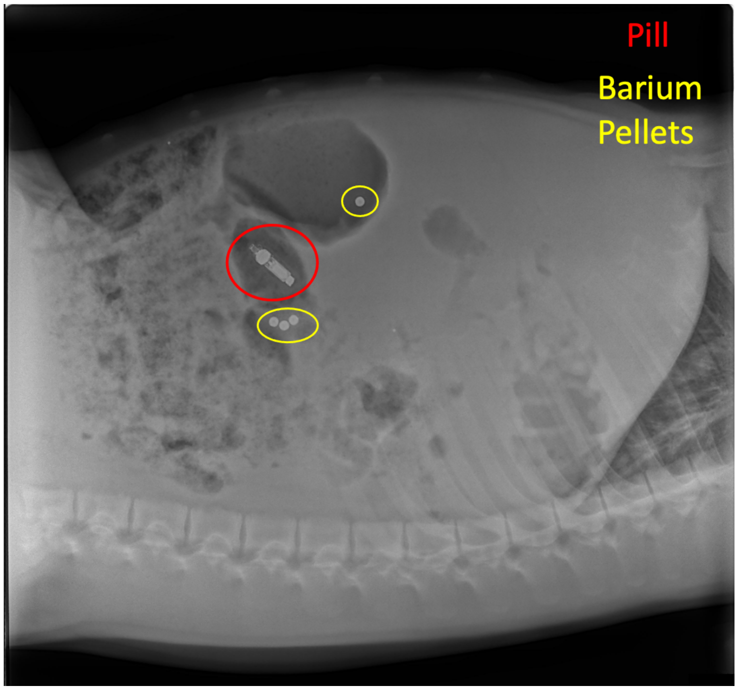

Supplemental Figure 9. Radiographic image of the VIBES pill and barium tracking pellets. The VIBES pill (encircled in red) and barium pellets (encircled in yellow) can be seen in the GI tract one day following oral administration in a swine in this radiographic image.

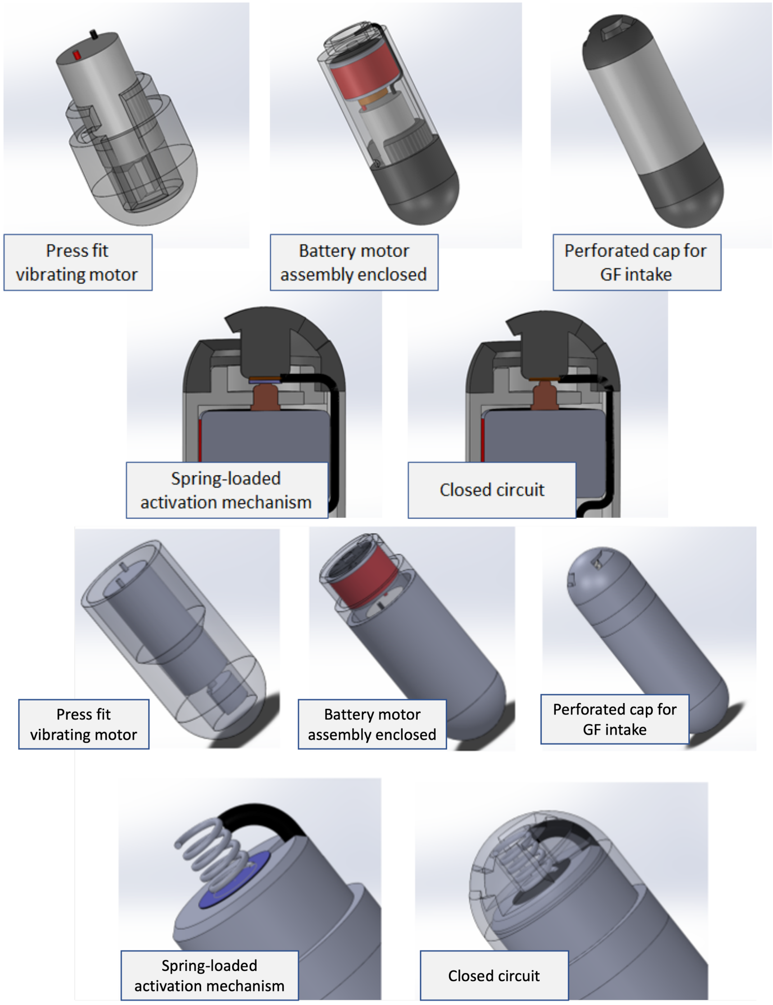

Supplemental Figure 10. Composition and Activation Mechanism of the VIBES pill.

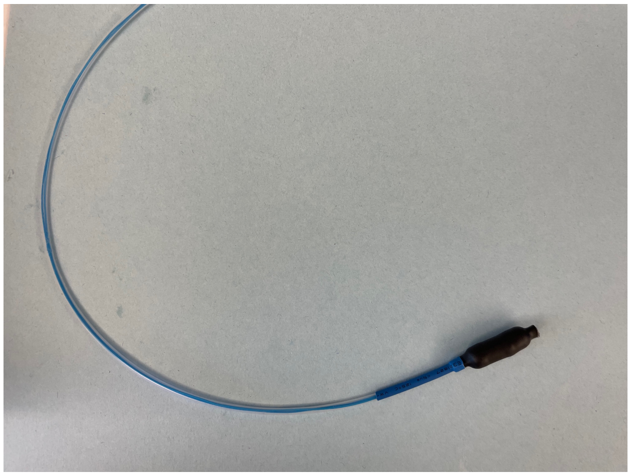

Supplemental Figure 11. Tethered VIBES pill for placement and maintenance through a PEG tube.

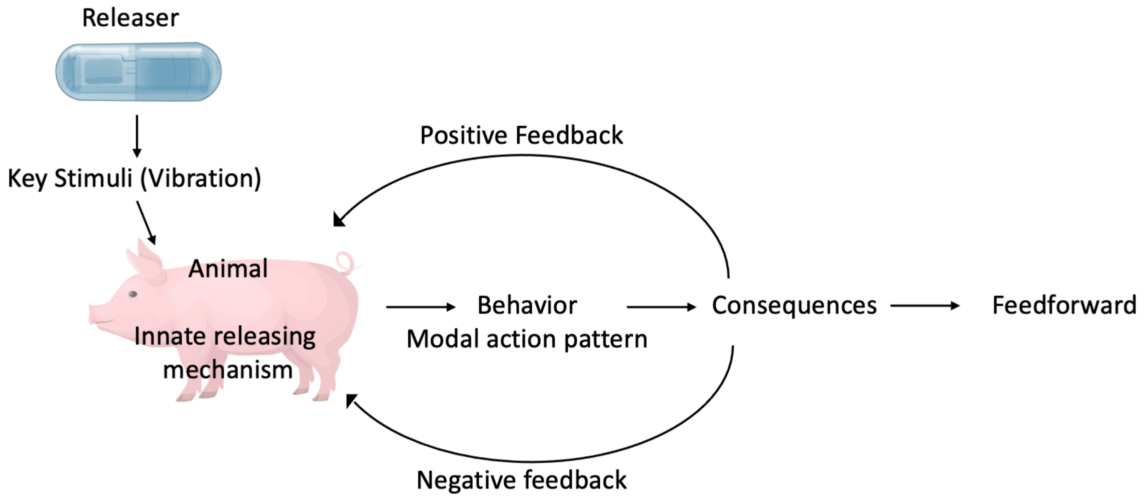

Supplemental Figure 12. Behavioral model, adapted from Lehner's model. Illustration by Virginia Fulford.

0 Data Collection

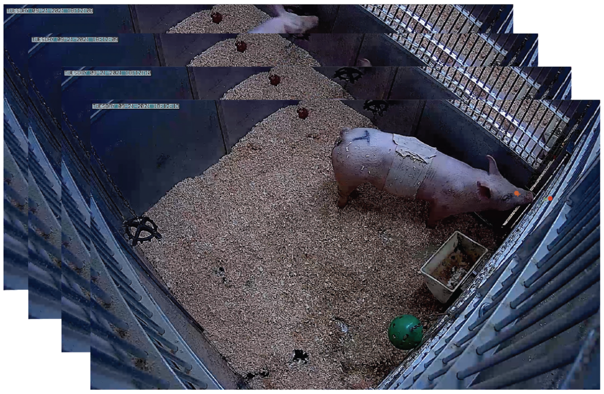

1 Detection and Tracking

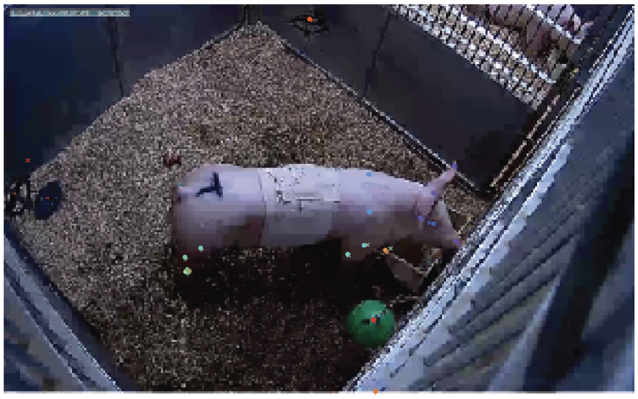

Description of Data:
- Collected between Sept 2021 - Feb 2022
- 24/7 data collection across 2 pens (1 camera each)
- 1-hour segments of continuous video and audio
- 1080p HD, night vision, 80 degree FOV
- Audio: 16000 Hz, mono, fltp, 15 kb/s
- Each file is approximately 400 Mb

Data Storage and Computing Clusters:
- AWS MIT Cloud Computing Servers (EC2)
- High throughput, low latency high performance computing clusters
- NVIDIA A100 Tensor Core GPUs

Training Data for Markerless Pose Estimation Model
- > 400 labeled images of body parts and objects in the environment.
- 2 camera perspectives
- Range of color data from RGB to B/W

Data Output
- Positional coordinate time-series data
- Coordinates of 22 body parts (e.g. snout) and 10 objects (e.g. gear toy)

2 Classification

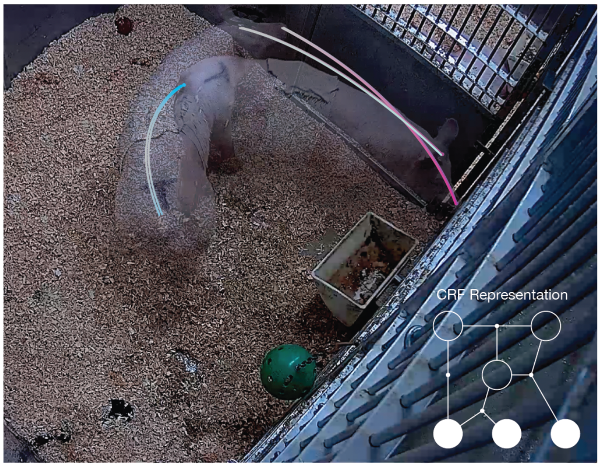

3 Behavior Characterization

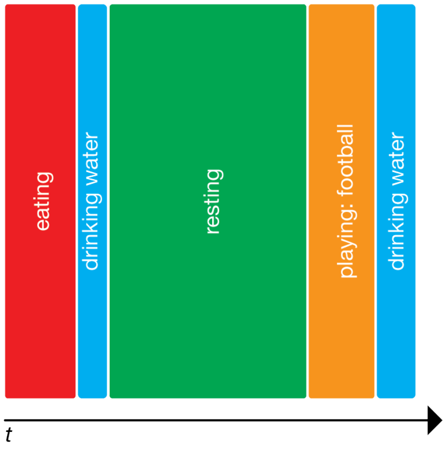

Data Manipulation
- From the positional coordinate time-series data of body parts, we calculate relative location at each time step.

Training Data
- Time-series data of the relative location of the head, snout, and tail (window = 39)

Data Output
- Estimated behaviors being conducted at time interval *t*

Plot the Estimated Behaviors
- Investigate inter- and intra-individual trends

Supplemental Figure 13. Behavioral model analysis pipeline.

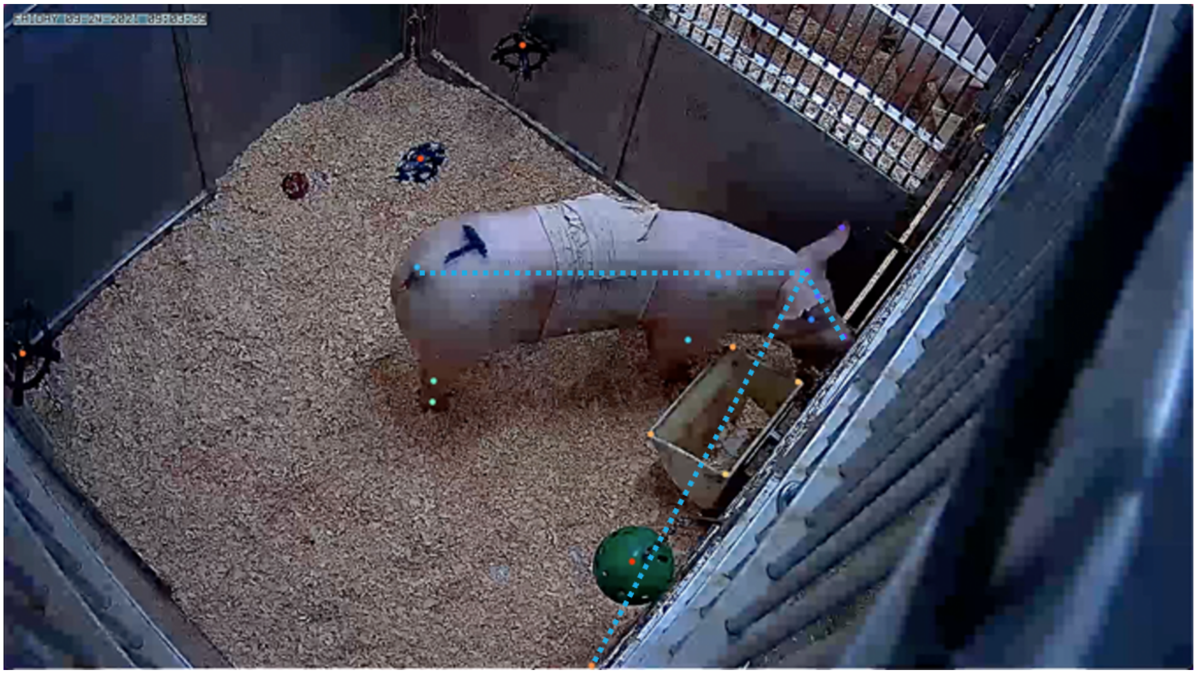

Supplemental Figure 14. Markless pose estimation: The generated features provide an estimate of body angle and distance

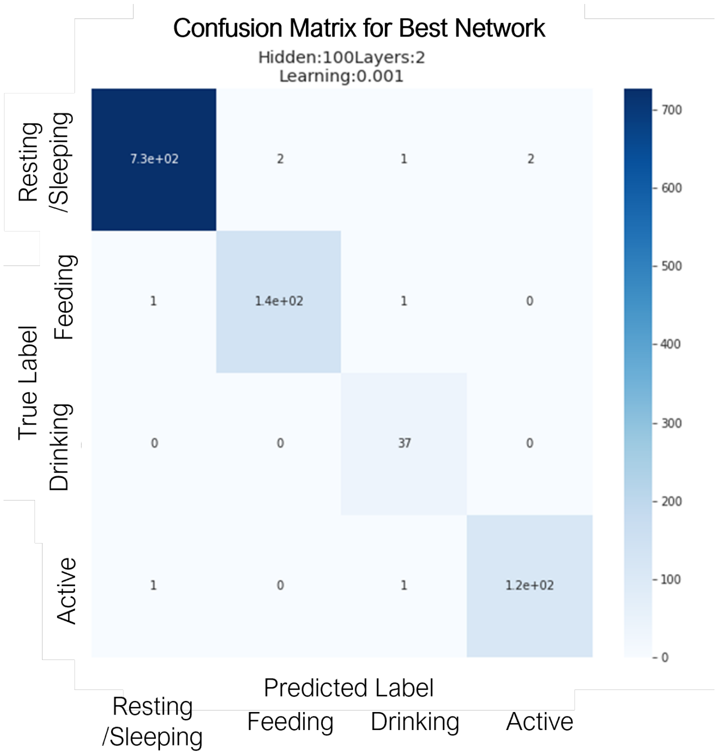

Supplemental Figure 15. Results of the LSTM prediction model

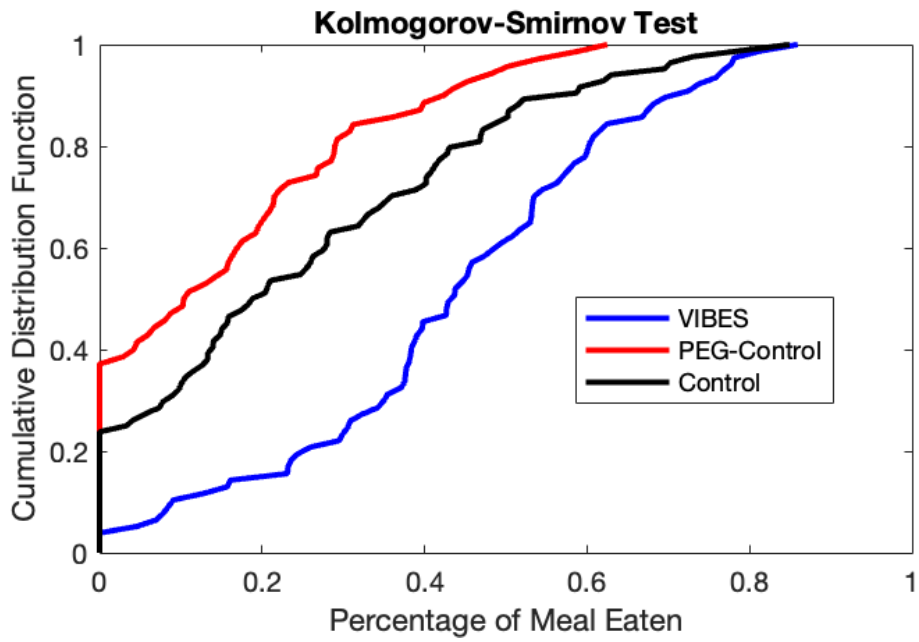

Supplemental Figure 16. Distribution of Intake Data. Comparison of three distributions of intake data using the Kolmogorov-Smirnov test. The blue, red, and black lines represent the empirical cumulative distribution functions of the VIBES, PEG-control, and control groups, respectively. The maximum vertical distance between the VIBES and Control lines (KS statistic = 0.6065) indicates a significant difference between the distributions (p = 1.1103e-12). The PEG-Control and Control distributions are not statistically significant p = 0.0555, KS statistic = 0.2119.

Supplemental Note 1:

Density of the pill was calculated using the properties of the following components:

Masses:

- Pill capsule: 1.17 g
- Motor (6x10): 1.6 g
- Battery: 1.2 g
- Pogo pin: 0.03 g
- Wire : 0.3 g
- Total mass: 4.3g

- Volume (11 mm diam pill) = 2.13 cm^3

- Pill density: 2.019 g/cm^3

Supplemental Table 1. Days required for passage of the barium pellets for animals orally administered a VIBES or sham pill.

	Number of Days to Pass all Barium Pellets	
Animal #	VIBES	Controls
1	4	9
2	5	7
3	4	9

Supplemental Table 2. The feature set for the keypoint model.

Animal or Object	Body Part Keypoints
Pig	Front Legs (elbow joint, carpal, and coffin), Rear Legs (stifle joint, tarsal, and coffin), Left and Right Scapula, Apex of Left and Right Ear, Head, Eyes, Snout, Proximal and Distal Tail Locations
Hopper	4 corners of the top of the hopper
Water Spigot	Spigot mouth
Toys	Center of the tethered and free moving toys

Supplemental Table 3. Data collection breakdown by subject

General Category	Behavior Type	Behavior Description
Inactive	Sleeping	No movement, not standing, eyes open
	Resting	No movement, not standing, eyes closed
Feeding	Free feeding	Head is in food bin, chewing
	Pedialyte	Pedialyte through cage doors while in-vivo team exchanges batteries / fixes bandages: head touching bottle
Drinking	Water Spigot	Head to water spigot
Playing	Hanging Toy Ball	Head or body touching the hanging ball
	Hanging gear	Head or body touching the gears
	Free moving toy	Interaction with head / body; moving
	Scratching / rooting woodchips	Pawing at or using their nose to move woodchips
	Scratching or climbing on walls of cage	Body up and touching the walls of the cage
	Interaction with researcher	Touching, nosing, being fed from researcher

Supplemental Video 1: Endoscopic visualization of the stomach during implantation of the VIBES tethered system.
Supplemental Video 2: Endoscopic visualization of the stomach after two weeks of treatment with the VIBES tethered system.
Supplemental Video 3: Endoscopic visualization of the stomach demonstrating the tethered VIBES system.
